# Eye‐Tracking Phenotypes Across the Depressive Spectrum: Joint Modeling of Diagnostic Group and Symptom Severity

**DOI:** 10.1002/cns.71198

**Published:** 2026-10-02

**Authors:** Xiangting Chen, Min Huang

**Affiliations:** ^1^ Department of General Medicine The Affiliated Suzhou Hospital of Nanjing Medical University (Suzhou Municipal Hospital) Suzhou Jiangsu China

**Keywords:** eye tracking, machine learning, major depressive disorder, subthreshold depression, symptom severity

## Abstract

**Aims:**

To characterize eye‐tracking alterations across the depressive spectrum and evaluate their value for diagnostic stratification and symptom severity estimation.

**Methods:**

In this cross‐sectional study, 183 participants completed a multi‐paradigm eye‐tracking battery. Group differences and associations with 17‐item Hamilton Depression Rating Scale (HAMD‐17) scores were examined with multiple‐comparison correction and covariate adjustment. Classification, regression, and multi‐task models used repeated nested resampling with fold‐specific preprocessing.

**Results:**

False discovery rate (FDR)‐significant between‐group differences were observed for two first‐fixation latency measures from lateral‐gaze orienting and for antisaccade accuracy. Among 35 candidate features, 33 had covariate‐adjusted group associations at *p* < 0.05. Across 539 retained features, covariate‐adjusted generalized additive models identified eight FDR‐significant associations with HAMD‐17. In multi‐task analysis, Route B had numerically higher mean internal performance than Route A, although paired between‐route differences were imprecisely estimated (accuracy, 0.929 vs. 0.864; macro F1, 0.928 vs. 0.859; R^2^, 0.834 vs. 0.596). Regression‐only performance was similar to Route B (R^2^, 0.847).

**Conclusion:**

Eye‐tracking alterations spanned the depressive spectrum and were associated with symptom severity after covariate adjustment. Between‐route differences were imprecisely estimated, and joint‐task training showed no clear improvement in severity estimation. External and temporal validation are required before clinical use.

## Graphical abstract

A multi‐paradigm eye‐tracking battery identified oculomotor alterations across normal controls, subthreshold depression, and major depressive disorder, particularly in lateral‐gaze orienting and antisaccade control. Hierarchical joint modeling showed numerically higher internal diagnostic performance, although route differences were imprecisely estimated, and eye‐tracking features were associated with symptom severity after covariate adjustment.

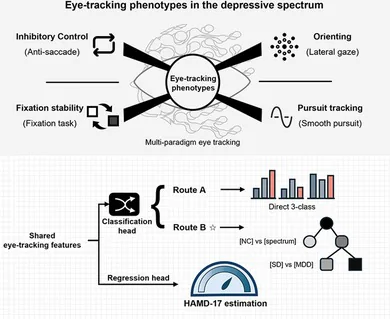

## 1 Introduction

Major depressive disorder (MDD) is a prevalent and disabling condition associated with substantial individual and societal burden. Although diagnosis is based on established clinical criteria, routine assessment still relies heavily on symptom reporting and clinician judgment, limiting the availability of objective tools for stratification and monitoring. Recent work in digital biomarkers and machine learning (ML) has therefore focused on behavioral signals that may complement conventional clinical assessment [1, 2, 3]. Subthreshold depression (SD) is of particular interest because it is common, clinically meaningful, and associated with increased risk of subsequent MDD [4, 5]. Epidemiologic and intervention studies support placing SD within the depressive spectrum [6, 7, 8, 9, 10].

Eye tracking has emerged as a promising approach in depression research because it captures visual orienting, sustained attention, inhibitory control, and broader oculomotor function [11]. Prior studies have reported abnormalities across free‐viewing, fixation, smooth‐pursuit, prosaccade, antisaccade, and facial emotion paradigms, including altered saccade dynamics, reduced gaze toward socially salient facial regions, and mood‐congruent gaze allocation [12, 13, 14, 15, 16, 17]. However, important gaps remain. Most studies have used binary case–control designs, single paradigms, or emotionally valenced stimuli. Studies explicitly including SD are limited, objective stratification tools across the depressive spectrum remain scarce, and joint modeling of diagnostic category and symptom severity has rarely been examined. Methodological reviews have also highlighted the need to control information leakage and better define what predictive models capture [2, 3].

In the present study, we used a multi‐paradigm eye‐tracking battery to examine normal controls (NC), SD, and MDD within the same analytic framework. We first assessed group differences and associations with depressive symptom severity at the feature level, followed by covariate‐adjusted analyses. We then evaluated classification and regression models under a repeated nested resampling framework with fold‐specific preprocessing. Finally, we developed a multi‐task framework to jointly estimate diagnostic group and symptom severity indexed by the 17‐item Hamilton Depression Rating Scale (HAMD‐17).

## 2 Methods

### 2.1 Study Population

Participants were recruited from Suzhou Municipal Hospital and Suyuan Community Health Service Station in Suzhou, China. The study included MDD, SD, and NC groups. Licensed psychiatrists assigned diagnostic groups after ICD‐11 assessment. Participants with MDD met ICD‐11 criteria for a current depressive episode and had HAMD‐17 scores greater than 17. Participants with SD had HAMD‐17 scores of 8–17 and no current ICD‐11 depressive episode, as confirmed by a psychiatrist. These score ranges were prespecified as adjuncts to clinical group assignment. NC participants had no current depressive disorder, HAMD‐17 scores of 7 or lower, and CES‐D scores below 16. CES‐D was used for baseline characterization and auxiliary symptom screening, not as a mandatory SD eligibility threshold. Clinical ratings and eye‐tracking assessments were completed on the same day. Diagnostic assessment and HAMD‐17 ratings were conducted by trained licensed psychiatrists, with senior psychiatrist review before dataset locking. Additional eligibility, recruitment, and assessment details are provided in Appendix A. The sample comprised eligible participants who completed both assessments during the recruitment period; no formal a priori sample‐size calculation was performed for the high‐dimensional prediction analyses.

### 2.2 Eye‐Tracking Assessment

Sociodemographic, lifestyle, and psychosocial data were collected using a standardized questionnaire. Eye‐tracking assessments were conducted at both recruitment sites using the same 60‐Hz Tobii Pro Nano device and a standardized acquisition protocol under controlled conditions. The battery comprised emotional‐word viewing, affective‐image free viewing, fixation stability, lateral gaze, smooth pursuit, prosaccade, and antisaccade tasks, covering attention, fixation control, orienting, pursuit, and inhibitory control (Appendix B).

### 2.3 Preprocessing and Feature Extraction

Raw gaze samples were segmented into fixations, saccades, and blinks using the Velocity‐Threshold Identification algorithm. Trial quality control used tracking ratio and task‐specific criteria. Task‐ and condition‐derived features were aggregated to 723 participant‐level variables. Feature‐level inference used label‐blind filtering based solely on missingness, variability, and inter‐feature correlation; diagnostic group and HAMD‐17 were not used in this reduction. Predictive modeling restarted from the 723‐variable matrix within each training partition (Appendix C).

### 2.4 Statistical Analysis

Baseline characteristics and eye‐tracking features were compared across groups using parametric or nonparametric methods as appropriate, with effect sizes reported for feature‐level analyses. Recruitment‐site distributions were compared using the Pearson χ^2^ test, and tracking accuracy and precision were compared between sites using two‐sided Mann–Whitney U tests. Associations with HAMD‐17 were assessed using Pearson and Spearman correlations and penalized generalized additive models (GAMs). A separate GAM was fitted for each of the 539 retained eye‐tracking features. Each model included a low‐dimensional penalized smooth for the eye‐tracking feature; primary models also included age, sex, and BMI. Benjamini–Hochberg false discovery rate correction was applied across all 539 smooth‐term tests. GAM robustness was assessed using stronger smoothing penalties, smooth‐term effective degrees of freedom, and Freedman–Lane residual permutation. Fitted smooths were characterized descriptively. All analyses were two‐sided and performed in Python and R; expanded statistical details are provided in Appendix F.

### 2.5 Predictive Modeling

Predictive models used five stratified outer train‐test repeats with five‐fold inner cross‐validation; each repeat allocated 85% of participants to training‐validation and 15% to held‐out testing. Preprocessing and feature retention were fitted only within training data. Label‐blind filtering preceded supervised selection using diagnostic group for classification and HAMD‐17 for regression. Fitted transformations and selected features were applied unchanged to validation and outer‐test data.

Classification used L1‐regularized multinomial logistic regression and random forest permutation importance for fold‐wise selection; regression used LASSO and random forest permutation importance. Candidate learners spanned regularized linear, kernel, tree‐ensemble, gradient‐boosting, and multilayer‐perceptron models. Ensembles included SuperLearner‐NNLS, ridge stacking, softmax weighting, and mean averaging.

Incremental value was assessed across clinical‐only, combined, and eye‐tracking‐only predictor sets, with symptom scales used for screening or outcome definition excluded. Performance was estimated by repeated nested resampling. No external or temporal validation was performed. The learner families, preprocessing rules, and ensemble strategies used in the repeated comparisons were specified before outer‐test evaluation. No single learner or ensemble was designated as primary, and model rankings were descriptive. For classification, TabM and xRFM were evaluated only in outer repeat 1 and were excluded from repeat‐level confidence‐interval estimation and calculation of the descriptive composite score (Appendix D).

### 2.6 Multi‐Task Modeling

A secondary multi‐task model jointly estimated diagnostic group and HAMD‐17 severity from eye‐tracking features. Route A used a three‐class softmax head; Route B first separated NC from the depressive spectrum and then SD from MDD. The same outer splits were used. Inner folds supported temperature calibration, and an internal validation subset guided early stopping. The architecture‐matched regression‐only sensitivity model retained the Route B encoders, quality‐informed gating, fused representation, regression architecture, preprocessing, outer splits, and training settings, while omitting the classification objective. Details are provided in Appendices E and F. Reporting followed the STROBE recommendations for cross‐sectional studies and the TRIPOD+AI recommendations for prediction‐model studies. Completed STROBE and TRIPOD+AI checklists are provided in Appendix G.

## 3 Results

### 3.1 Baseline Characteristics

The analytic cohort included 183 participants, with 61 in each group. Suzhou Municipal Hospital contributed 50 NC, 50 SD, and 51 MDD participants, while Suyuan Community Health Service Station contributed 11 NC, 11 SD, and 10 MDD participants. Recruitment‐site distribution was similar across groups (Pearson χ^2^ = 0.076, *p* = 0.963). Age approached imbalance across groups (Kruskal–Wallis H = 5.896, *p* = 0.053), with median age declining from 55 years in NC to 44 years in SD and 41 years in MDD. Sex, education, marital status, and living arrangement showed no significant group differences (Table 1). Clinical ratings differed across groups, with stepwise increases in the Center for Epidemiologic Studies Depression Scale, HAMD‐17, the 7‐item Generalized Anxiety Disorder Scale, and the 14‐item Hamilton Anxiety Rating Scale from NC to SD to MDD. Employment type, income satisfaction, sleep status, physical exercise, social activities, and emotional disclosure also differed across groups, whereas smoking and alcohol use did not. BMI was modestly lower in MDD than in SD.

**TABLE 1:** Baseline demographic and clinical characteristics of the study groups.

Characteristic	NC (*n* = 61)	SD (*n* = 61)	MDD (*n* = 61)	Test statistic	*p*
Recruitment site, *n* (%)				0.076	0.963
Suzhou Municipal Hospital	50 (81.97%)	50 (81.97%)	51 (83.61%)		
Suyuan Community Health Service Station	11 (18.03%)	11 (18.03%)	10 (16.39%)		
Age, years	55.00 (31.00, 66.00)	44.00 (30.00, 60.00)	41.00 (29.00, 57.00)	5.896	0.053
Sex, *n* (%)				2.603	0.272
Female	39 (63.93%)	44 (72.13%)	47 (77.05%)		
Male	22 (36.07%)	17 (27.87%)	14 (22.95%)		
Education level, *n* (%)				7.170	0.518
Compulsory education	17 (27.87%)	12 (19.67%)	12 (19.67%)		
Basic education	8 (13.11%)	14 (22.95%)	16 (26.23%)		
Vocational education	1 (1.64%)	4 (6.56%)	3 (4.92%)		
Professional education	30 (49.18%)	25 (40.98%)	27 (44.26%)		
Postgraduate education	5 (8.20%)	6 (9.84%)	3 (4.92%)		
Employment type, *n* (%)				13.312	0.038
Mainly manual labor	9 (14.75%)	13 (21.31%)	11 (18.03%)		
Mixed manual and mental labor	14 (22.95%)	18 (29.51%)	17 (27.87%)		
Mainly mental labor	35 (57.38%)	29 (47.54%)	23 (37.70%)		
Unemployed	3 (4.92%)	1 (1.64%)	10 (16.39%)		
Satisfaction with current income, *n* (%)				29.093	< 0.001^a^, ^b^, ^c^
Dissatisfied	6 (9.84%)	8 (13.11%)	22 (36.07%)		
Neutral	26 (42.62%)	40 (65.57%)	30 (49.18%)		
Satisfied	29 (47.54%)	13 (21.31%)	9 (14.75%)		
Marital status, *n* (%)				9.077	0.169
Married	44 (72.13%)	42 (68.85%)	38 (62.30%)		
Unmarried	13 (21.31%)	18 (29.51%)	17 (27.87%)		
Divorced	0 (0%)	0 (0%)	3 (4.92%)		
Widowed	4 (6.56%)	1 (1.64%)	3 (4.92%)		
Children, *n* (%)				3.052	0.217
With children	46 (75.41%)	41 (67.21%)	37 (60.66%)		
Without children	15 (24.59%)	20 (32.79%)	24 (39.34%)		
Living arrangement, *n* (%)				0.919	0.632
Living alone	11 (18.03%)	14 (22.95%)	10 (16.39%)		
Not living alone	50 (81.97%)	47 (77.05%)	51 (83.61%)		
Height, m	1.63 (1.58, 1.70)	1.62 (1.60, 1.68)	1.60 (1.60, 1.66)	0.248	0.883
Weight, kg	58.00 (53.50, 67.00)	55.00 (52.00, 65.00)	54.00 (50.00, 63.00)	5.486	0.064
BMI, kg/m^2^	22.72 (20.55, 24.24)	21.74 (19.92, 23.01)	20.82 (19.53, 22.84)	6.565	0.038^c^
Sleep status, *n* (%)				72.604	< 0.001^a^, ^b^, ^c^
Normal	38 (62.30%)	14 (22.95%)	4 (6.56%)		
Poor sleep quality	21 (34.43%)	26 (42.62%)	15 (24.59%)		
Insomnia	2 (3.28%)	21 (34.43%)	42 (68.85%)		
Dietary pattern, *n* (%)				26.830	< 0.001^b^, ^c^
Mainly vegetarian	4 (6.56%)	2 (3.28%)	20 (32.79%)		
Balanced	51 (83.61%)	55 (90.16%)	38 (62.30%)		
Mainly meat‐based	6 (9.84%)	4 (6.56%)	3 (4.92%)		
Smoking status, *n* (%)				4.336	0.631
Never	53 (86.89%)	52 (85.25%)	49 (80.33%)		
Occasional	1 (1.64%)	3 (4.92%)	5 (8.20%)		
Regular	3 (4.92%)	4 (6.56%)	5 (8.20%)		
Quit	4 (6.56%)	2 (3.28%)	2 (3.28%)		
Alcohol use, *n* (%)				7.499	0.277
Never	46 (75.41%)	48 (78.69%)	37 (60.66%)		
Occasional	11 (18.03%)	9 (14.75%)	18 (29.51%)		
Regular	2 (3.28%)	2 (3.28%)	5 (8.20%)		
Quit	2 (3.28%)	2 (3.28%)	1 (1.64%)		
Physical exercise, *n* (%)				39.369	< 0.001^a^, ^b^, ^c^
Never	12 (19.67%)	29 (47.54%)	45 (73.77%)		
Occasional	22 (36.07%)	20 (32.79%)	10 (16.39%)		
Regular	27 (44.26%)	12 (19.67%)	6 (9.84%)		
Cognitive activities, *n* (%)				21.467	< 0.001^a^, ^b^
Never	22 (36.07%)	37 (60.66%)	47 (77.05%)		
Occasional	25 (40.98%)	15 (24.59%)	8 (13.11%)		
Regular	14 (22.95%)	9 (14.75%)	6 (9.84%)		
Housework, *n* (%)				7.367	0.118
None	2 (3.28%)	9 (14.75%)	11 (18.03%)		
Partial	35 (57.38%)	34 (55.74%)	31 (50.82%)		
All	24 (39.34%)	18 (29.51%)	19 (31.15%)		
Social activities, *n* (%)				66.556	< 0.001^a^, ^b^, ^c^
None	5 (8.20%)	32 (52.46%)	49 (80.33%)		
1–3/month	41 (67.21%)	21 (34.43%)	10 (16.39%)		
4–6/month	8 (13.11%)	3 (4.92%)	0 (0%)		
≥ 6/month	7 (11.48%)	5 (8.20%)	2 (3.28%)		
Emotional disclosure, *n* (%)				70.854	< 0.001^a^, ^b^, ^c^
None	4 (6.56%)	31 (50.82%)	50 (81.97%)		
Occasional	39 (63.93%)	21 (34.43%)	9 (14.75%)		
Frequent	18 (29.51%)	9 (14.75%)	2 (3.28%)		
Tracking accuracy	0.67 (0.54, 0.96)	0.78 (0.54, 1.08)	0.69 (0.49, 1.19)	2.046	0.360
Tracking precision	0.50 (0.29, 0.90)	0.72 (0.44, 1.31)	0.74 (0.34, 1.26)	4.625	0.099
CES‐D score	4.00 (2.00, 9.00)	13.00 (10.00, 17.00)	21.00 (18.00, 26.00)	105.268	< 0.001^a^, ^b^, ^c^
HAMD‐17 total score	2.00 (1.00, 3.00)	10.00 (9.00, 12.00)	19.00 (18.00, 21.00)	162.460	< 0.001^a^, ^b^, ^c^
GAD‐7 score	2.00 (0.00, 3.00)	9.00 (6.00, 12.00)	12.00 (8.00, 15.00)	99.817	< 0.001^a^, ^b^, ^c^
HAMA‐14 score	2.00 (1.00, 3.00)	9.00 (5.00, 12.00)	14.00 (9.00, 16.00)	103.631	< 0.001^a^, ^b^, ^c^

*Note:* Continuous variables are presented as median (interquartile range) and were compared using the Kruskal–Wallis test, followed by Dunn tests with Bonferroni correction. Categorical variables are presented as *n* (%) and were compared using the χ^2^ or Fisher exact test, as appropriate. Superscripts denote significant pairwise differences after correction.

Abbreviations: NC, normal control; SD, subthreshold depression; MDD, major depressive disorder; BMI, body mass index; CES‐D, Center for Epidemiologic Studies Depression Scale; HAMD‐17, 17‐item Hamilton Depression Rating Scale; GAD‐7, 7‐item Generalized Anxiety Disorder Scale; HAMA‐14, 14‐item Hamilton Anxiety Rating Scale.

^a^ NC versus SD.

^b^ NC versus MDD.

^c^ SD versus MDD. Tracking accuracy and precision are eye‐tracking data‐quality measures.

### 3.2 Eye‐Tracking Data Quality

Tracking accuracy and precision were similar across diagnostic groups (Table 1) and recruitment sites (Mann–Whitney U tests, *p* = 0.902 and *p* = 0.417, respectively). Figure S1 summarizes preprocessing, and Figures S2–S3 show fixation‐density maps.

### 3.3 Feature‐Level Group Differences

Label‐blind quality control and redundancy reduction yielded 539 features for group comparisons. Thirty‐five showed nominal differences and were retained for descriptive follow‐up (Table S1). BH‐FDR correction identified three features. Both first‐fixation latency measures from the lateral‐gaze orienting task were longer in SD and MDD than in NC. Antisaccade accuracy decreased from NC to SD to MDD. Among the 35 candidate features, 33 had nominal covariate‐adjusted group associations at *p* < 0.05. The largest adjusted effects involved the two lateral‐gaze latency measures and antisaccade accuracy, followed mainly by area‐of‐interest visit‐ and fixation‐duration measures from lateral gaze, sinusoidal pursuit, and fixation stability (Figure S4).

### 3.4 Associations With Symptom Severity

Across the 539 retained eye‐tracking features, nominal associations with HAMD‐17 were observed for 30 features by Pearson correlation and 13 by Spearman correlation, but none survived BH‐FDR correction. Unadjusted GAMs identified 93 features with FDR‐significant smooth terms. In the primary covariate‐adjusted analysis of all 539 features, eight associations remained FDR‐significant. They involved antisaccade accuracy, two lateral‐gaze first‐fixation latency measures, three visit‐duration measures from fixation‐stability interference, lateral‐gaze saccadic peak‐velocity variability, and lateral‐gaze mean visit duration (Table S2A–B).

Adjusted partial R^2^ ranged from 0.089 to 0.204, with a median smooth‐term EDF of 2.208. The fitted smooths were predominantly increasing for the two lateral‐gaze first‐fixation latency measures and predominantly decreasing for the remaining six associations (Figure S5). All eight associations remained FDR‐significant under the stronger smoothing penalties (λ = 2 and λ = 5) and were supported by Freedman–Lane permutation analyses (all empirical *p* < 0.001; all BH‐FDR q < 0.001; Table S2B–C).

### 3.5 Classification Modeling

Classification feature selection combined L1‐regularized multinomial logistic regression and random forest permutation importance within each outer training–validation partition. Across five outer repeats, 53–63 features were selected; 41 appeared in all repeats, and the mean pairwise Jaccard index was 0.800. Selected features spanned lateral gaze, smooth pursuit, fixation stability, antisaccade, and prosaccade domains (Figure S6; Tables S3–S5). Mean internal outer‐test performance was similar for XGBoost, LightGBM, and CatBoost (Figure 1). Accuracy ranged from 0.900 to 0.907, macro F1 from 0.899 to 0.908, and macro‐AUROC from 0.973 to 0.984. These model comparisons were descriptive. Hyperparameter settings and inner‐loop performance are reported in Tables S6 and S7. Mean differences between ensemble strategies were more evident for macro‐AUROC and log loss than for threshold‐based metrics. Compared with mean averaging, SuperLearner‐NNLS and softmax weighting had mean macro‐AUROC values of 0.982 versus 0.972 and mean log‐loss values of 0.260 and 0.258 versus 0.515, respectively (Figure S8). Ensemble configurations and weights are reported in Table S8 and Figure S7.

**FIGURE 1:**
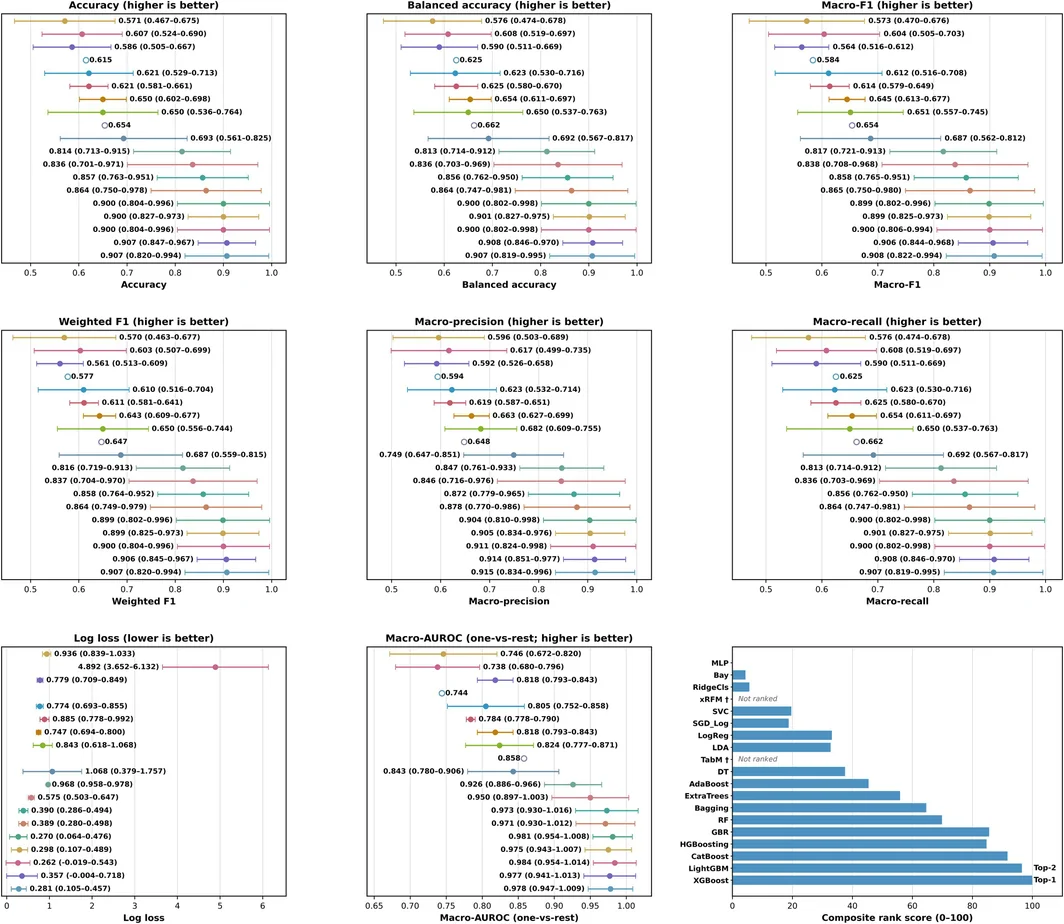
Internal outer‐test classification performance and descriptive composite scoring of candidate models using eye‐tracking features. Points and error bars show mean performance and repeat‐level 95% confidence intervals across five held‐out outer‐test splits. The composite score summarizes metric‐specific ranks on a 0–100 scale. TabM and xRFM were evaluated only in outer repeat 1 and are shown as single‐execution references. They were excluded from confidence‐interval estimation and calculation of the descriptive composite score. †Single‐execution outer‐test reference model. Confidence intervals were calculated on the original metric scale and were not truncated to metric bounds. AUROC, area under the receiver operating characteristic curve.

In predictor‐set comparisons, adding eye‐tracking features increased mean performance relative to the corresponding clinical‐only models across all three clinical‐variable layers. The combined‐basic, combined‐lifestyle, and combined‐extended models achieved accuracy values of 0.879 ± 0.082, 0.886 ± 0.081, and 0.893 ± 0.094, respectively, with macro‐AUROC values of 0.985 ± 0.016, 0.982 ± 0.019, and 0.981 ± 0.018. With SuperLearner‐NNLS, paired accuracy gains ranged from 0.264 to 0.471 and macro‐AUROC gains from 0.174 to 0.408; the corresponding repeat‐level 95% confidence intervals excluded zero (Table S9). With symptom scales excluded from the predictor sets, these estimates indicate incremental internal predictive information beyond the prespecified non‐scale clinical‐variable layers. Confusion matrices for SuperLearner‐NNLS showed mean recall of 89% for NC, 89% for SD, and 100% for MDD, with no direct confusion between NC and MDD (Figure S9). Interpretability analyses identified lateral‐gaze and pursuit‐related saccadic measures, together with stability‐related latency features, as the leading classification contributors (Figures S10–S12).

### 3.6 Regression Modeling

HAMD‐17 regression combined LASSO and random forest permutation importance within each outer training–validation partition. Across five repeats, 46–55 features were selected; 42 appeared in all repeats, and the mean pairwise Jaccard index was 0.818. Retained features included antisaccade accuracy, lateral‐gaze first‐fixation latency, and fixation‐duration measures in saccade paradigms (Figure S13; Tables S3 and S10).

Mean internal outer‐test performance was similar for LightGBM and XGBoost (Figure 2). MAE was below 2.0, RMSE was approximately 2.5, and R^2^ ranged from 0.885 to 0.886. Learning‐based ensembles showed numerically lower mean prediction errors and higher mean R^2^ than mean averaging. Ensemble configurations and performance comparisons are reported in Table S12 and Figures S14 and S15. Hyperparameter settings and inner‐loop performance are provided in Tables S6 and S11.

**FIGURE 2:**
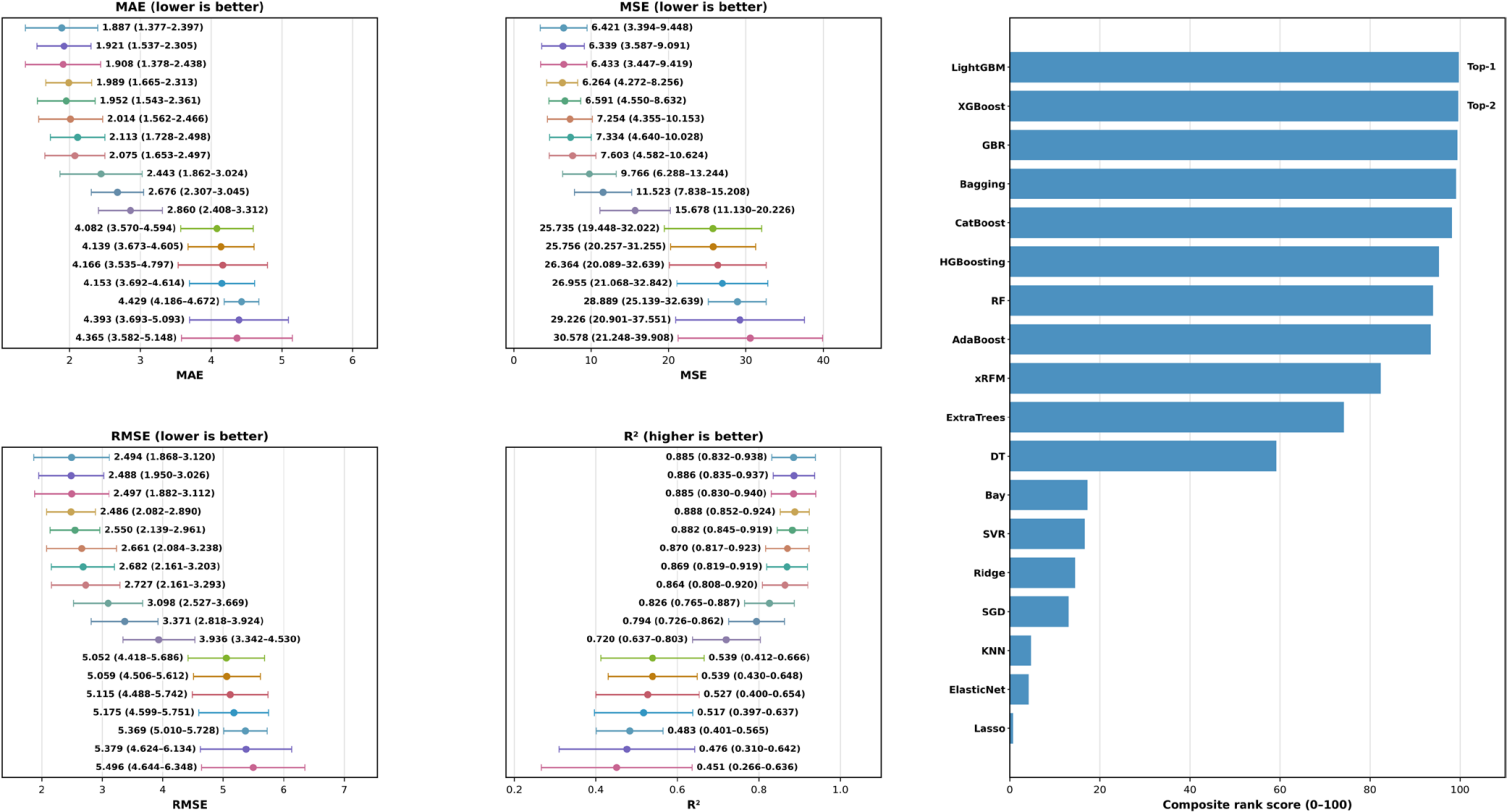
Internal outer‐test regression performance and descriptive composite scoring of individual candidate models for HAMD‐17 estimation using eye‐tracking features. Points and error bars show mean performance and repeat‐level 95% confidence intervals across five held‐out outer‐test splits. The composite score averages min–max‐normalized R^2^ and reverse‐coded MAE, MSE, and RMSE on a 0–100 scale; higher values indicate better performance. No confidence interval was calculated for the composite score. HAMD‐17, 17‐item Hamilton Depression Rating Scale; MAE, mean absolute error; MSE, mean squared error; RMSE, root mean squared error; R^2^, coefficient of determination.

In predictor‐set comparisons, adding eye‐tracking features reduced mean prediction error and increased mean R^2^ relative to the corresponding clinical‐only models across all three clinical‐variable layers. With SuperLearner‐NNLS, R^2^ ranged from 0.217 ± 0.099 for the clinical‐basic model to 0.558 ± 0.062 for the clinical‐extended model. The combined‐basic, combined‐lifestyle, and combined‐extended models achieved R^2^ values of 0.885 ± 0.037, 0.887 ± 0.036, and 0.893 ± 0.026, respectively. Paired R^2^ gains ranged from 0.335 to 0.668, and MAE reductions ranged from 2.328 to 3.435 points; the corresponding repeat‐level 95% confidence intervals excluded zero (Table S13). Figures S16–S18 show observed–predicted agreement and interpretability results, with leading contributions arising mainly from saccadic and smooth‐pursuit dynamics and showing complex dependence patterns.

### 3.7 Multi‐Task Modeling

Preprocessing‐derived feature retention was relatively consistent across outer repeats. Fold‐wise preprocessing retained 516 to 546 features, with 21 to 22 retained task‐domain blocks. Among 560 unique retained features, 500 were retained in all five repeats, and the mean pairwise Jaccard index was 0.951. Domain‐level gating weights showed a distributed profile, with relatively higher weights assigned mainly to smooth‐pursuit and lateral‐gaze blocks, while fixation‐stability blocks also ranked among the higher‐weighted domains in Route A (Tables S3 and S14–S16).

Route B had numerically higher mean outer‐test classification performance than Route A (accuracy, 0.929 vs. 0.864; macro F1, 0.928 vs. 0.859; log loss, 0.220 vs. 0.292), although the paired repeat‐level 95% confidence intervals for accuracy and macro F1 included zero (Figure 3; Table S17). Misclassification under Route A was concentrated in SD. Route B retained 100% recall for NC and reduced SD‐to‐NC misclassification without reducing MDD recall (Figure 4A–B).

**FIGURE 3:**
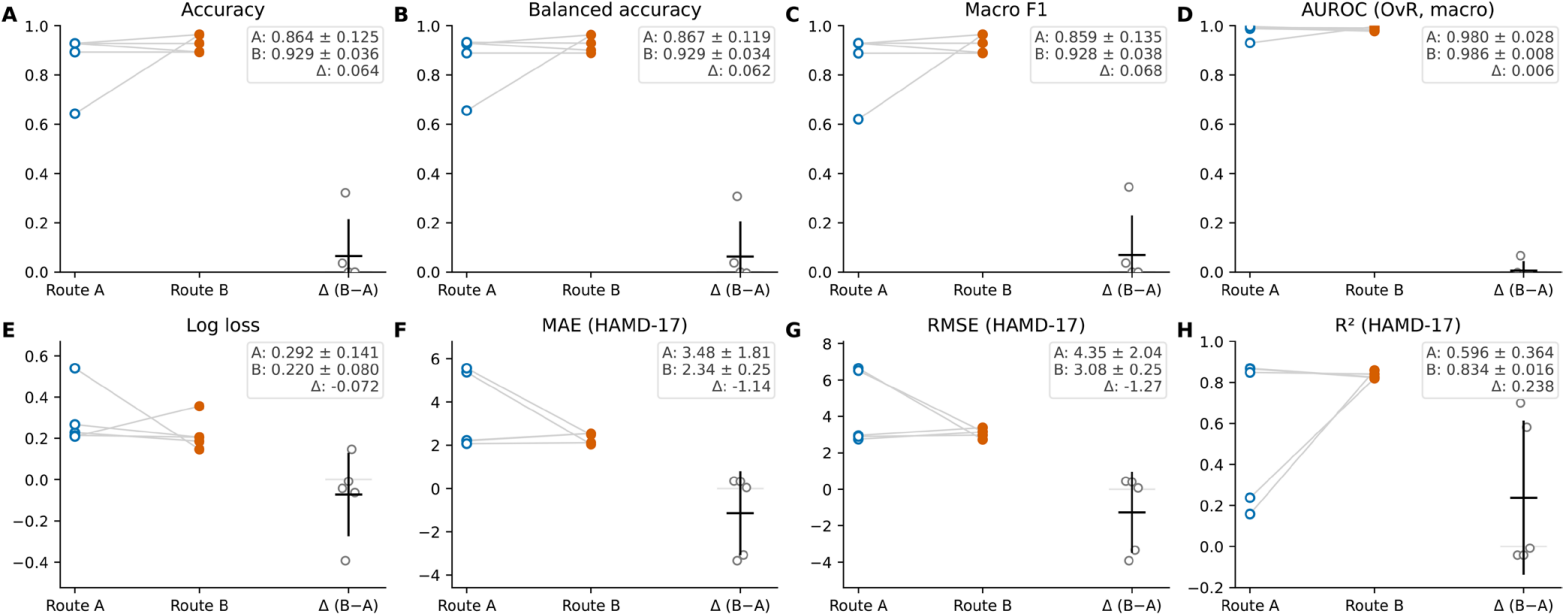
Internal outer‐test performance of the eye‐tracking multi‐task model under Route A and Route B. Points represent five matched outer‐test splits, with gray lines connecting results from the same split. Panels A–E show classification performance, and panels F–H show HAMD‐17 prediction. Insets report mean ± standard deviation and paired Route B‐minus‐Route A differences. AUROC, area under the receiver operating characteristic curve; OvR, one‐versus‐rest; macro, macro‐averaged.

**FIGURE 4:**
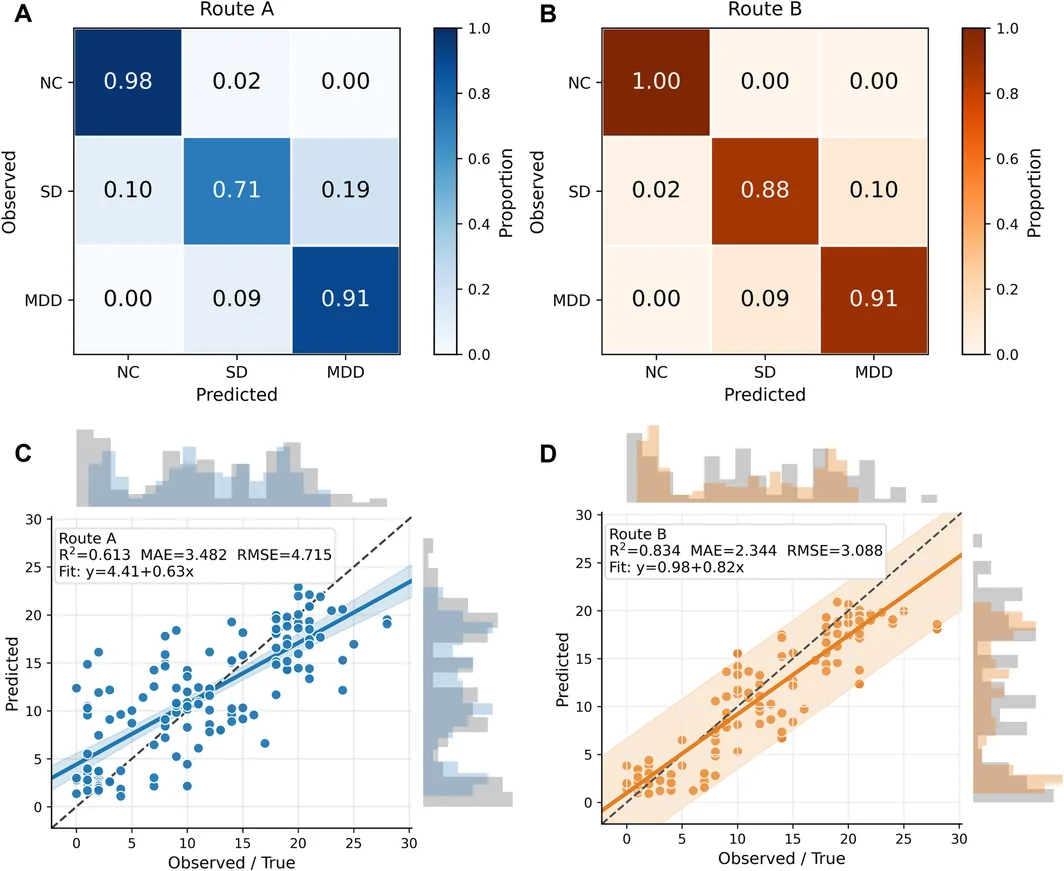
Diagnostic error patterns and agreement between observed and predicted HAMD‐17 under Route A and Route B. Panels A and B show row‐normalized confusion matrices for three‐class diagnosis. Panels C and D show observed and predicted HAMD‐17 scores from pooled out‐of‐sample predictions, together with fitted regression lines and the line of identity. Performance statistics in Panels C and D were recalculated after pooling predictions across outer repeats and may differ from mean repeat‐level estimates. MAE, mean absolute error; RMSE, root mean squared error; R^2^, coefficient of determination; NC, normal control; SD, subthreshold depression; MDD, major depressive disorder.

For HAMD‐17 estimation, Route B had numerically lower mean MAE and RMSE and higher mean R^2^ than Route A, with mean R^2^ values of 0.834 and 0.596, respectively. The paired R^2^ difference was 0.238 (95% CI, −0.222 to 0.698). Pooled out‐of‐sample predictions under Route A underestimated higher symptom levels, whereas Route B more closely tracked the observed range (Figure 4C–D; Figure S19). The regression‐only analysis yielded a mean outer‐test R^2^ of 0.847 (repeat‐level 95% CI, 0.815–0.879), compared with 0.834 (0.814–0.853) for Route B. The paired R^2^ difference was −0.013 (95% CI, −0.050 to 0.023), with similarly small paired differences in MAE and RMSE (Table S17). Figure 5 shows hierarchical probabilities and domain‐gating profiles for three illustrative cases.

**FIGURE 5:**
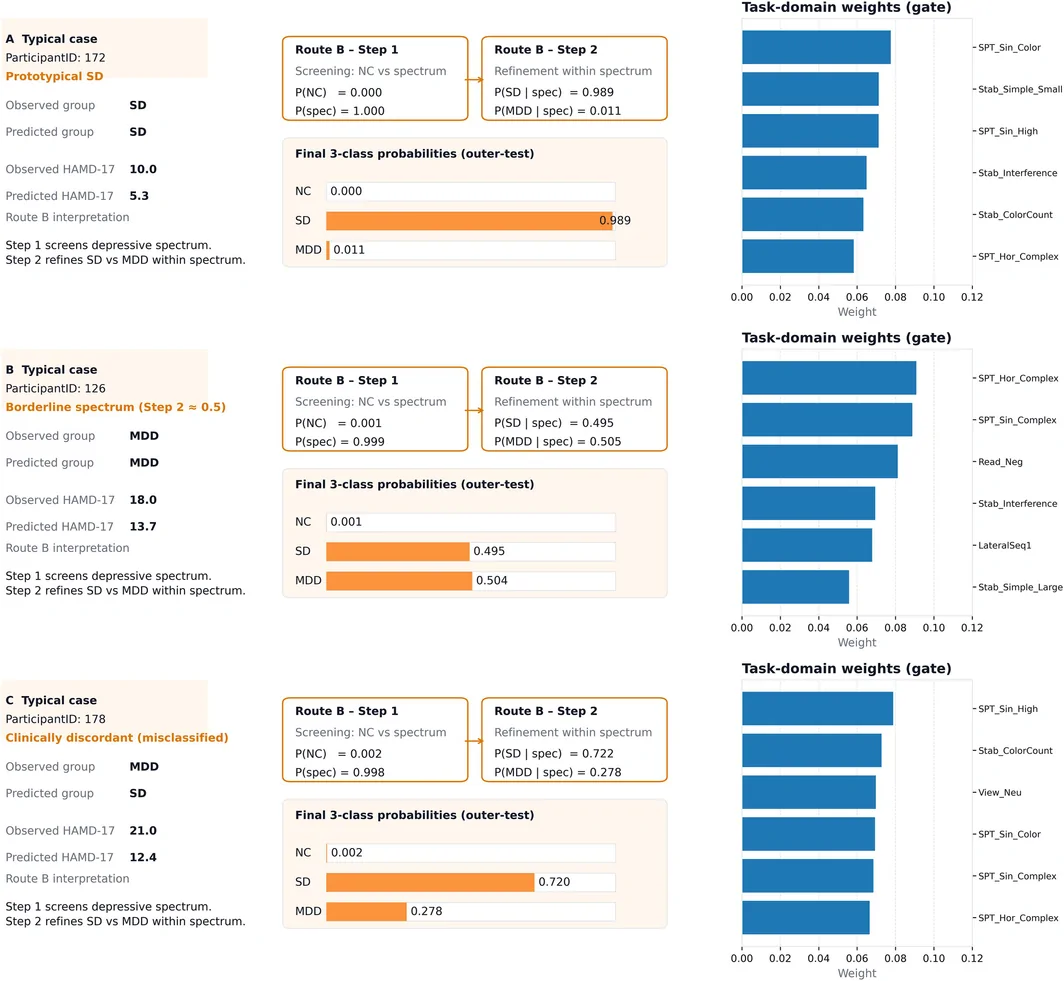
Individual‐level clinical interpretation of Route B within a two‐step hierarchical diagnostic framework. Three illustrative participants from the outer‐test sets are shown. The left panels present observed and predicted diagnostic groups and HAMD‐17 scores. The middle panels show the two‐stage hierarchical probabilities and derived three‐class probabilities. The right panels show task‐domain gating weights. NC, normal control; SD, subthreshold depression; MDD, major depressive disorder; HAMD‐17, 17‐item Hamilton Depression Rating Scale.

## 4 Discussion

In this two‐site cohort, eye‐tracking features differed across NC, SD, and MDD and showed GAM‐based associations with HAMD‐17 severity. These features also supported categorical classification and dimensional severity estimation within an internal resampling framework. The most robust group‐level differences involved first‐fixation latency during lateral‐gaze orienting and antisaccade accuracy, implicating attentional and oculomotor‐control processes beyond emotion‐related gaze allocation. Route B had numerically higher mean internal performance, particularly for SD recognition, although paired between‐route estimates were imprecise.

The prominence of first‐fixation latency in the lateral‐gaze task and antisaccade accuracy is clinically plausible. First‐fixation latency indexes the efficiency of orienting toward a newly relevant peripheral target, whereas antisaccade performance depends on the ability to inhibit a prepotent reflexive response and generate a goal‐directed saccade. Together, these findings implicate inefficiency in attentional allocation and top‐down inhibitory control, with possible involvement of frontoparietal and frontostriatal systems supporting executive and saccadic control. This interpretation is consistent with prior reports of oculomotor abnormalities across prosaccade, antisaccade, free‐viewing, and smooth‐pursuit paradigms and with evidence that coordinated eye‐movement patterns carry clinically informative signals [15, 16, 18, 19, 20, 21, 22, 23]. Several key features differed in SD and showed a graded pattern across NC, SD, and MDD. Given the association of SD with subsequent MDD [6, 7, 8, 9, 10], prospective studies should examine whether these eye‐tracking measures predict clinical progression [24, 25]. Definitions of SD vary across epidemiologic, clinical, and intervention studies [5]. In this study, licensed psychiatrists assigned diagnostic status according to ICD‐11, and SD required the absence of a current depressive episode together with a HAMD‐17 score of 8–17. The HAMD‐17 provided a standardized clinician‐rated measure of symptom severity [26]. This study‐specific definition should be considered in cross‐study comparisons.

Correlation analyses yielded no FDR‐significant associations. Covariate‐adjusted GAMs identified eight associations after correction across all 539 features. These involved antisaccade accuracy, two lateral‐gaze first‐fixation latency measures, fixation‐stability visit‐duration measures, and lateral‐gaze visit‐duration and saccadic‐velocity measures. The fitted smooths generally increased for the two first‐fixation latency measures and decreased for the remaining associations. Modest effective degrees of freedom and concordant results under stronger smoothing penalties and Freedman–Lane permutation testing supported the stability of the estimates. A significant GAM smooth term alone does not demonstrate nonlinearity. Curve shapes were reported descriptively, and independent replication is needed.

Prior studies have linked depression severity to dysphoric attentional maintenance, reduced gaze toward positive or socially salient regions, and mood‐congruent gaze allocation [11, 27, 28, 29, 30, 31]. In this cohort, the FDR‐significant GAM associations arose primarily from lateral‐gaze, fixation‐stability interference, and antisaccade tasks. The associated measures included orienting latency, inhibitory control, visit duration, and saccadic variability. These results extend depressive eye‐tracking phenotypes to visuomotor and executive‐control processes alongside affective attentional bias.

Internal performance was similar across several tree‐based boosting models and ensembles. Predictor‐set comparisons indicated incremental information from eye‐tracking features beyond prespecified non‐scale clinical variables. Within this internal‐validation framework, eye tracking may provide an adjunctive signal for depressive‐spectrum phenotyping. The models remain investigational and should be interpreted within clinician‐led assessment. Repeated nested resampling, fold‐specific preprocessing, and outer‐repeat stability analyses reduced bias from information leakage, data‐dependent preprocessing, and unstable feature selection. Residual overfitting remains possible because the feature space was high dimensional relative to the sample size. External and temporal validation is required before performance can be assessed across sites, devices, and clinical populations. Recent methodological reviews similarly emphasize external validation, missingness handling, and clear separation between exploratory and confirmatory modeling pipelines [3, 32, 33, 34, 35].

The interpretability analyses attributed the largest contributions to lateral‐gaze, pursuit, and stability‐related features, with smaller contributions from emotional‐word and affective‐image tasks. This distribution is consistent with behavioral digital biomarker studies reporting clinically relevant information across multiple modalities and temporal scales [22, 23, 36, 37, 38, 39, 40]. Prior studies have shown that eye movements can support depression classification and severity estimation in single‐task settings [14, 15, 16, 17, 20, 22, 23], while joint modeling remains less studied. Route B had numerically higher mean classification performance than Route A, particularly for SD, although the repeat‐level confidence intervals were wide. The architecture‐matched regression‐only model performed similarly to Route B (paired R^2^ difference, −0.013; 95% CI, −0.050 to 0.023), providing no clear evidence that the classification objective improved HAMD‐17 estimation. Because HAMD‐17 bands contributed to group assignment, validation with independently defined diagnostic and dimensional outcomes remains necessary.

Several limitations warrant consideration. The cross‐sectional design and recruitment from two sites in one city limit longitudinal inference and transportability, and no external or temporal validation cohort was available. The feasibility‐based cohort was modest relative to the initial feature space, and no formal a priori sample‐size calculation was performed for the high‐dimensional prediction analyses. Performance estimates therefore remain imprecise despite stratified nested resampling and fold‐specific model development. The sample size also precluded reliable subgroup fairness analyses and site‐specific performance estimates. The study‐specific SD definition, combining psychiatrist‐confirmed absence of a current ICD‐11 depressive episode with HAMD‐17 scores of 8–17, may limit comparability with studies using other definitions [5, 26]. Psychotropic medication exposure was limited and heterogeneous, and information on class, dose, duration, adherence, and timing was incomplete. Employment type and income satisfaction also differed across groups but were outside the prespecified adjustment set. Residual confounding by medication exposure and socioeconomic factors remains possible, particularly for latency‐sensitive and saccade‐based measures. Future studies should collect detailed treatment and socioeconomic data and, where feasible, include medication‐naive or treatment‐stratified cohorts. The GAM‐based severity analyses were exploratory. Although eight associations survived full‐family covariate adjustment and were supported under stronger smoothing penalties and Freedman–Lane permutation testing, the fitted smooths were estimated within the same cohort and may remain sample‐dependent. Most showed predominantly monotonic patterns, and no formal test of departure from linearity was undertaken. Curve‐shape descriptors should therefore not be interpreted as evidence of specific nonlinear mechanisms or clinically meaningful thresholds. Independent replication is needed to establish the stability and clinical relevance of these severity associations. The 60 Hz sampling rate may introduce temporal quantization in latency‐sensitive and short‐duration saccadic measures. These measures should be interpreted as task‐level oculomotor indices. Finally, the absence of psychiatric comparison groups limits conclusions regarding diagnostic specificity, given partially overlapping eye‐movement abnormalities across affective disorders [13].

In summary, eye‐tracking measures, particularly lateral‐gaze first‐fixation latency and antisaccade accuracy, differed across NC, SD, and MDD and showed covariate‐adjusted associations with symptom severity. Prediction performance was estimated within this cohort and requires confirmation in multicenter, device‐independent, and longitudinal studies before clinical or community use.

## Funding

This study was supported by the Jiangsu Provincial Medical Key Discipline (ZDXK202252), Jiangsu Provincial Health Commission Key Medical projects (K2023014), and Suzhou Gusu Health Talent Plan Talent Research Project (GSWS2022068). The funders had no role in the study design or implementation, data collection, management, analysis, interpretation, manuscript preparation, review, approval, or the decision to submit the manuscript for publication.

## Ethics Statement

The study protocol was approved by the Ethics Committee of Suzhou Municipal Hospital, China (approval No. K‐2025‐278‐K01), and the study was conducted in accordance with the Declaration of Helsinki, as revised in Brazil in 2013. Written informed consent was obtained from all participants before enrollment.

## Conflicts of Interest

The authors declare no conflicts of interest.

## Supporting information


**Figure S1:** Eye‐tracking preprocessing and analysis pathways.
**Figure S2:** Spatial distribution of gaze during lateral gaze and smooth pursuit tasks.
**Figure S3:** Spatial distribution of attention to facial stimuli across emotional valence conditions.
**Figure S4:** Top 20 covariate‐adjusted eye‐tracking features across diagnostic groups.
**Figure S5:** Covariate‐adjusted GAM associations for the eight FDR‐significant eye‐tracking features identified across all 539 retained features.
**Figure S6:** Supervised eye‐tracking feature selection for three‐class classification in outer repeat 1.
**Figure S7:** Base‐learner weight distributions across outer repeats for the NNLS Super Learner and softmax‐weighted ensembles.
**Figure S8:** Outer‐test performance of ensemble models relative to the mean‐averaging baseline.
**Figure S9:** Confusion matrices of SuperLearner‐NNLS across five outer repeats and the mean matrix in the eye‐tracking modality.
**Figure S10:** Global SHAP feature contributions for the SuperLearner‐NNLS ensemble in the eye‐tracking modality.
**Figure S11:** SHAP beeswarm and dependence plots for the SD class in the eye‐tracking modality.
**Figure S12:** Individual‐level LIME explanations for SuperLearner‐NNLS predictions in the eye‐tracking modality.
**Figure S13:** Supervised eye‐tracking feature selection for HAMD‐17 regression in outer repeat 1.
**Figure S14:** Base‐learner weight profiles and across‐repeat stability of ensemble models for HAMD‐17 regression in the eye‐tracking modality.
**Figure S15:** Outer‐test performance differences between learning‐based ensembles and mean averaging for HAMD‐17 regression.
**Figure S16:** Agreement between observed and predicted HAMD‐17 scores on outer test sets across ensemble strategies.
**Figure S17:** Global SHAP feature importance and effect patterns for the eye‐tracking regression model.
**Figure S18:** Individual‐level LIME explanations for HAMD‐17 regression across severity tertiles.
**Figure S19:** Residual diagnostics for HAMD‐17 prediction under Route A and Route B.
Appendix A. Eligibility criteria, group definitions, recruitment, and medication exposure.
Appendix B. Eye‐tracking task battery and acquisition details.
Appendix C. Eye‐tracking preprocessing and feature extraction.
Appendix D. Predictive modeling details.
Appendix E. Multi‐task model details.
Appendix F. Expanded statistical analysis and reporting details.
Appendix G. Reporting guideline checklists.

**Table S1:** Group comparisons and covariate‐adjusted analyses of candidate eye‐tracking features associated with depressive states.
**Table S2:** GAM‐based associations between eye‐tracking features and HAMD‐17 severity: full‐family covariate‐adjusted analysis and sensitivity analyses.
**Table S3:** Outer‐repeat stability of feature selection and feature retention across predictive modeling components.
**Table S4:** Outer‐repeat supervised feature‐selection stability for the single‐task classification model.
**Table S5:** Task‐domain distribution of selected classification features by outer‐repeat selection frequency.
**Table S6:** Selected hyperparameter settings across outer training–validation sets.
**Table S7:** Inner cross‐validation performance of classification models using eye‐tracking features.
**Table S8:** Stability of ensemble configurations across outer repeats in the eye‐tracking modality.
**Table S9:** Internal incremental predictive value of eye‐tracking features for three‐class classification beyond prespecified clinical‐variable layers.
**Table S10:** Outer‐repeat stability of supervised feature selection for the HAMD‐17 regression model.
**Table S11:** Inner‐loop cross‐validation performance of regression models for HAMD‐17 prediction using eye‐tracking features.
**Table S12:** Stability of ensemble configurations across outer repeats for HAMD‐17 regression in the eye‐tracking modality.
**Table S13:** Internal incremental predictive value of eye‐tracking features for HAMD‐17 estimation beyond prespecified clinical‐variable layers.
**Table S14:** Outer‐repeat feature‐retention and task‐domain stability in the multi‐task model.
**Table S15:** Largest task‐domain blocks retained after multi‐task preprocessing.
**Table S16:** Domain‐level gating‐weight profiles across outer repeats in the multi‐task model.
**Table S17:** Repeat‐level performance uncertainty and architecture‐matched regression‐only sensitivity analysis of the multi‐task model.

## Data Availability

The data that support the findings of this study are available on request from the corresponding author. The data are not publicly available due to privacy or ethical restrictions.
