# Supplementary material for: Eye‐Tracking Phenotypes Across the Depressive Spectrum: Joint Modeling of Diagnostic Group and Symptom Severity
Source: CNS Neurosci Ther. 2026 Oct 2;32(10):e71198. doi: 10.1002/cns.71198 (PMC13632823; doi:10.1002/cns.71198)
Supplement: Supplementary file 1 [file CNS-32-e71198-s001.docx]

**Supplementary Material**

**Supplementary Appendix A. Eligibility criteria, group definitions, recruitment, and medication exposure**

A1. Eligibility criteria

Eligibility criteria were age 18–80 years, either sex, at least primary school education, and adequate Mandarin reading ability to complete the tasks. Participants were required to be able to complete clinical assessment and eye-tracking testing on the same day.

A2. Exclusion criteria

Participants were excluded if they had severe medical illness likely to affect participation, schizophrenia spectrum disorders, bipolar disorder, depressive episodes with psychotic symptoms, intellectual disability, impaired consciousness, or substance abuse or dependence. Pregnancy and lactation were exclusionary. Recent acute infection, fever, or marked stress responses were excluded. Conditions likely to compromise eye-tracking validity were also exclusionary, including clinically significant visual or hearing impairment, high myopia, color blindness, and ophthalmic diseases affecting visual function.

A3. Group definitions

Group definitions were prespecified before analysis. Clinical diagnostic status was established by licensed psychiatrists using ICD-11 criteria. MDD participants met ICD-11 criteria for a current depressive episode and had HAMD-17 scores greater than 17. SD participants had clinically relevant depressive symptoms on standardized clinical assessment, HAMD-17 scores of 8–17, and psychiatrist-confirmed absence of a current ICD-11 depressive episode. NC participants had no current depressive disorder, HAMD-17 scores of 7 or lower, and CES-D scores below 16.

HAMD-17 was used to standardize clinician-rated symptom severity across the three groups. ICD-11 assessment established the presence or absence of a current depressive episode. The CES-D contributed to baseline characterization and auxiliary symptom screening; SD eligibility followed the clinical and HAMD-17 criteria described above. Group assignments were reviewed by a senior psychiatrist before dataset locking, with uncertain cases resolved through consensus.

A4. Diagnostic assessment and rater procedures

Diagnostic assessment and HAMD-17 administration were performed by licensed psychiatrists who completed standardized training before participant enrollment. Training included ICD-11 diagnostic criteria, HAMD-17 item anchors, standardized interview procedures, and calibration with training cases. Group assignment was reviewed by a senior psychiatrist before dataset locking, and cases with uncertain diagnostic status or HAMD-17 scoring were adjudicated through consensus discussion. Clinical assessors did not have access to processed eye-tracking features or model outputs during diagnostic assessment and HAMD-17 scoring.

A5. Psychotropic medication exposure

Psychotropic medication use did not constitute an exclusion criterion. Psychotropic medication exposure was recorded descriptively when available, including previous antidepressant exposure and medications used for sleep symptoms. Exposure was limited and heterogeneous, and information on medication class, dose, duration, adherence, and timing relative to eye-tracking assessment was incomplete. Psychotropic medication exposure was therefore not suitable for reliable medication-based adjustment or stratified analysis.

A6. Recruitment period and sample-size considerations

Participants were enrolled and assessed between January and June 2025. The sample size reflected the number of eligible participants who completed clinical and eye-tracking assessments during the recruitment period. A formal a priori sample-size calculation was not undertaken for the high-dimensional prediction analyses. Balanced enrollment across the three groups facilitated stratified resampling, although the cohort remained modest relative to the initial predictor space.

A7. Protocol, registration, and patient and public involvement

No formal prospective prediction-model protocol was publicly archived, and the study was not registered. Patients and members of the public were not involved in the design, conduct, reporting, or dissemination of the study.

**Supplementary Appendix B. Eye-tracking task battery and acquisition details**

B1. Acquisition setting

At both recruitment sites, eye tracking was conducted in a quiet room under controlled conditions using the same Tobii Pro Nano eye tracker and standardized acquisition procedures. The device sampled gaze at 60 Hz and was mounted beneath a 14.5-inch display with a resolution of 1920 × 1080 pixels. Viewing distance was maintained at 60 to 70 cm. A 9-point calibration was completed before the task battery and repeated as needed to achieve acceptable calibration quality. Standardized instructions were provided before each task. Practice trials were administered when applicable. A 30-second rest interval followed each task. At a sampling frequency of 60 Hz, the nominal inter-sample interval was approximately 16.7 ms. Latency- and saccade-duration-related features were therefore interpreted as approximate task-level behavioral indices rather than high-resolution saccadic measures. This constraint was considered most relevant to saccade onset, saccade offset, latency, and short-duration saccadic measures, whereas fixation-duration, gaze-allocation, and aggregate task-level measures were considered less sensitive to the sampling interval.

B2. Task battery

The task battery comprised seven paradigms, with gaze recorded continuously throughout testing.

a. Emotional word viewing task.

Participants viewed Chinese words with positive, neutral, and negative valence presented on the screen. Stimuli were drawn from an established affective lexicon. Features were derived by valence condition where applicable.

b. Free viewing of affective images.

Participants viewed affective images presented in random order. Inter-stimulus intervals included a central fixation point. Viewing was unrestricted.

c. Fixation stability task.

Participants fixated a central target under three conditions: a simple condition, a distractor condition, and a color-counting condition.

d. Lateral gaze orienting task.

Participants shifted gaze from a central fixation point to peripheral targets presented in two prespecified orders. Each order was repeated twice. Each target was displayed for 3 s.

e. Smooth pursuit task.

Participants tracked a moving target under horizontal and sinusoidal motion profiles. Each profile included low-frequency motion at 0.2 Hz, high-frequency motion at 0.4 Hz, a distractor-background condition, and a color-change condition with silent counting. Horizontal pursuit trials lasted 10 s, and sinusoidal pursuit trials lasted 15 s.

f. Prosaccade task.

A central fixation point was presented for 2 s, followed by a 1 s gap. A peripheral target then appeared in one of four directions for 2 s. Seven trials were administered.

g. Antisaccade task.

Timing matched the prosaccade task. Participants were instructed to suppress a reflexive saccade toward the target and instead shift gaze to the mirror location. Seven trials were administered.

B3. Functional coverage of the task battery

The battery covered multiple oculomotor domains relevant to depressive psychopathology, including attentional allocation, fixation control, orienting latency, pursuit tracking, and inhibitory control.

**Supplementary Appendix C. Eye-tracking preprocessing and feature extraction**

Raw gaze samples were segmented into fixations, saccades, and blinks using the Velocity-Threshold Identification (I-VT) algorithm. Samples falling outside the stimulus display area were removed before event classification. An angular velocity threshold of 30°/s was used to distinguish saccades from fixations.

Trial-level data quality was assessed using the tracking ratio, defined as the proportion of valid gaze samples within a trial. Trials with a tracking ratio below 50% were excluded.

For saccade-based paradigms, additional validity criteria were applied. Because gaze was sampled at 60 Hz, saccade onset and offset times were resolved at the sample-grid level, and latency- and duration-based saccadic features were treated as approximate task-level indices. Trial inclusion required a correct response direction together with plausible latency. Prosaccade trials were retained when latency was between 80 and 800 ms. Antisaccade trials were retained when the response direction was correct and latency was at least 130 ms.

After quality control, eye-tracking features were computed from fixation and saccade events. Features were derived by task and condition, including valence-specific conditions where applicable, and then aggregated to the participant level.

After trial-level quality control and participant-level aggregation, the initial eye-tracking matrix comprised 723 variables. Two analytically distinct preprocessing pathways were used.

For feature-level inferential analyses, a label-blind quality-control and redundancy-reduction procedure was applied to the full analytic predictor matrix. Features with more than 20% missingness were excluded (n=139), no feature was excluded at the coefficient-of-variation threshold of 0.01, and 45 features were removed through correlation-based redundancy reduction at an absolute correlation threshold of 0.95. Diagnostic group, HAMD-17 score, inferential P values, and effect sizes were not used in this procedure. The resulting 539 variables constituted the feature-level inferential testing set.

Predictive modeling restarted from the original 723-variable matrix within each resampling training partition. Preprocessing parameters and retained feature indices were estimated from the training data only and applied unchanged to the corresponding validation or held-out outer-test data. Model-specific supervised feature selection, when used, was performed only after label-blind preprocessing and was restricted to the corresponding training partition.

**Supplementary Appendix D. Predictive modeling details**

D1. Repeated nested resampling framework

Model performance was evaluated using repeated nested resampling. In each of five outer repeats, participants were stratified by diagnostic group and split into an 85% training-validation set and a 15% held-out test set. Within each outer training-validation set, a stratified 5-fold inner cross-validation was used for hyperparameter tuning and, where applicable, ensemble-weight estimation.

All operations requiring parameter estimation were confined to the corresponding training partition and then applied unchanged to the paired validation fold or held-out outer test set. These operations included preprocessing, feature selection, model tuning, and ensemble fitting. The outer test set was not used for any model-development step. This framework was used to estimate internal out-of-sample performance only. Because no independent external or temporal validation cohort was available, these estimates should not be interpreted as evidence of model transportability.

D2. Fold-wise preprocessing

Predictive modeling restarted from the full 723-variable participant-level feature matrix within each resampling training partition. Prespecified label-blind preprocessing included missing-data handling, outlier truncation, scaling, low-variability filtering, and correlation-based redundancy reduction. All transformation parameters and feature-retention decisions were estimated using the corresponding training data only and were applied unchanged to the paired validation fold or held-out outer-test set. Diagnostic group and HAMD-17 were not used in this preprocessing stage.

For the single-task models, outcome-guided feature selection was subsequently performed within the same training partition. Diagnostic group was used for supervised classification feature selection, whereas HAMD-17 was used for supervised regression feature selection. Validation and outer-test outcomes were not used in these decisions.

D3. Supervised feature selection for classification

Supervised feature selection was embedded within the resampling workflow. L1-regularized multinomial logistic regression was tuned using macro-averaged F1 and used to retain features with non-zero coefficients. Random forest (RF) permutation importance was computed within the same training partition, and the top 25 ranked features were retained. Within inner cross-validation, the feature set for each validation fold was defined as the union of the two selectors fitted on the corresponding inner training fold. For outer-test evaluation, the same preprocessing and feature-selection workflow was refitted on the full outer training-validation set, and the resulting selected feature set was applied unchanged to the held-out outer test set.

D4. Classification models and ensemble strategies

A prespecified set of base classifiers was evaluated, including regularized linear models, kernel methods, tree-based ensembles, gradient-boosting models, and multilayer perceptrons. Hyperparameters were tuned within inner cross-validation using macro-averaged F1 as the primary criterion, and log loss was recorded to assess probabilistic fit.

Ensembles were built from inner-fold out-of-fold class probabilities using four strategies:

a. Super Learner with a non-negative least squares meta-learner (SuperLearner-NNLS)

b. Ridge stacking, using an L2-regularized multinomial meta-learner

c. Softmax weighting, using fold-aggregated log loss to derive model weights

d. Mean averaging, assigning equal weights across base learners

For outer test evaluation, base learners were refit on the full outer training-validation data with tuned hyperparameters. Ensemble weights and meta-learner parameters were then applied to the outer test set without re-estimation. No probability calibration was used in the single-task classification analyses. Predicted class labels were assigned to the class with the highest predicted probability. No post hoc decision-threshold optimization was performed. TabM and xRFM were each evaluated once on the held-out test set from outer repeat 1 and are presented in Figure 1 as supplementary outer-test references; they were excluded from repeat-level confidence intervals and composite ranking.

D5. Regression models and ensemble strategies

HAMD-17 total score was predicted under the same repeated nested resampling and fold-wise preprocessing framework. A prespecified set of base regressors was evaluated, including regularized linear models, kernel regression, tree-based ensembles, gradient-boosting regressors, and multilayer perceptrons. Hyperparameters were tuned using the coefficient of determination (R^2^), while mean absolute error and root mean squared error were also recorded.

Regression ensembles were constructed from out-of-fold point predictions using the same four strategies as in classification:

a. Super Learner with a non-negative least squares meta-learner

b. Ridge stacking with an L2-regularized linear meta-learner

c. Softmax weighting using fold-aggregated RMSE

d. Mean averaging

Base regressors were refit on the full outer training-validation data, and ensemble parameters were applied to the outer test set without re-estimation. The candidate classifier and regressor families, preprocessing rules, and ensemble strategies were specified before evaluation of held-out outer-test performance. No single learner or ensemble was designated as primary. Rankings were descriptive, and results were reported for all successfully evaluated configurations.

D6. Outer-repeat feature-selection stability analyses

We evaluated outer-repeat stability of supervised feature selection for the single-task classification and HAMD-17 regression models. In each outer repeat, the preprocessing and supervised selection workflow was restarted from the 723-variable eye-tracking feature matrix, fitted within the outer training-validation set, and used to record the final selected feature set. For classification, the final selected set was defined as the union of features retained by L1-regularized multinomial logistic regression and RF permutation importance. For regression, the final selected set was defined as the union of LASSO-selected features and the top-ranked RF permutation-importance features.

For each model, we summarized the number of selected features per outer repeat, the number of unique features selected at least once, the number of persistent features selected in all five outer repeats, the number of robust features selected in at least four outer repeats, and the pairwise Jaccard index between selected feature sets. The task-domain distribution of selected classification features was also summarized. These analyses are reported in Supplementary Tables S3–S5 and S10.

D7. Incremental classification-value analysis

To evaluate whether eye-tracking features provided predictive information beyond conventional clinical-demographic variables, predictor-set comparisons were conducted for three-class diagnostic classification within the same repeated nested resampling framework as the primary classification analysis.

Clinical predictors were organized into three prespecified layers. The basic layer included sex, age, religion, education, occupation, work type, income satisfaction, marital status, children, living arrangement, height, weight, and BMI. The lifestyle layer additionally included dietary pattern, smoking history, alcohol use, physical exercise, cognitive activities, and housework. The extended layer additionally included sleep status, social activity, and emotional disclosure.

CES-D and HAMD-17 were excluded from all classification predictor sets to avoid symptom-scale leakage, because HAMD-17 was used for clinician-rated severity stratification and CES-D was used for control-group symptom screening and baseline depressive-symptom characterization. GAD-7, HAMA-14, and anxiety status were excluded from the primary clinical layers to avoid symptom-scale dominance. Tracking accuracy and tracking precision were not included as clinical predictors because they reflected eye-tracking data quality. Accordingly, the clinical-only classification models served as prespecified non-scale clinical-variable baselines for estimating the incremental internal predictive information contributed by eye-tracking features.

For each clinical layer, clinical-only models were compared with combined models that incorporated fold-wise selected eye-tracking features. Candidate eye-tracking features were selected within each outer training-validation set using the same L1-regularized multinomial logistic regression and random forest permutation-importance union procedure as the primary eye-tracking classification analysis. Model tuning, ensemble construction, and outer-test evaluation followed the same repeated nested resampling framework as the primary classification analysis.

Paired gains were calculated within matched outer test splits. For ACC, BACC, F1, precision, recall, and macro-AUROC, gains were calculated as combined minus clinical-only. For log loss, gains were calculated as clinical-only log loss minus combined-model log loss, so positive values indicated improved probabilistic performance. Eye-tracking-only results from the primary classification analysis were retained as a reference rather than as a paired comparator.

D8. Incremental regression-value analysis

For HAMD-17 severity estimation, predictor-set comparisons were conducted within the same repeated nested resampling framework as the primary HAMD-17 regression analysis. The outcome was HAMD-17 total score. HAMD-17 was therefore not included as a predictor.

Clinical predictors were organized into the same three prespecified layers used in the classification predictor-set comparison. The basic layer included sex, age, religion, education, occupation, work type, income satisfaction, marital status, children, living arrangement, height, weight, and BMI. The lifestyle layer additionally included dietary pattern, smoking history, alcohol use, physical exercise, cognitive activities, and housework. The extended layer additionally included sleep status, social activity, and emotional disclosure.

CES-D, GAD-7, HAMA-14, anxiety status, and diagnostic group were not included in the clinical predictor sets. Tracking accuracy and tracking precision were also excluded because they reflected eye-tracking data quality rather than clinical predictors. Accordingly, the clinical-only regression models served as prespecified non-scale clinical-variable baselines for estimating the incremental internal predictive information contributed by eye-tracking features to HAMD-17 severity estimation.

For each clinical layer, clinical-only models were compared with combined models that incorporated fold-wise selected eye-tracking features. Candidate eye-tracking features were selected within each outer training-validation set using the same least absolute shrinkage and selection operator/random forest (LASSO/RF) union procedure as the primary HAMD-17 regression analysis. Model tuning, ensemble construction, and outer-test evaluation followed the same repeated nested resampling framework as the primary regression analysis.

Paired gains were calculated within matched outer test splits. For MAE, MSE, and RMSE, gains were calculated as clinical-only minus combined-model values, so positive values indicated error reduction. For R^2^, gains were calculated as combined minus clinical-only, so positive values indicated improved explained variance. Eye-tracking-only results from the primary HAMD-17 regression pipeline were retained as a reference and were not used as paired comparators.

**Supplementary Appendix E. Multi-task model details**

E1. Model overview

In a secondary analysis, we developed an eye-tracking-based multi-task model for joint diagnostic stratification and HAMD-17 severity estimation. Diagnostic labels were NC, SD, and MDD.

Eye-tracking variables were assigned to prespecified task domains on the basis of feature-name identifiers. These domains covered fixation stability, lateral gaze orienting, horizontal smooth pursuit, sinusoidal smooth pursuit, prosaccade, antisaccade, emotional word reading, and free viewing.

Each broader domain comprised one or more task-condition blocks defined by paradigm, sequence, load, or interference condition. Each domain was encoded into a fixed-dimensional embedding. Domain embeddings were then combined using a gating mechanism informed by domain-level data completeness. The resulting fused representation was passed to task-specific output heads.

E2. Domain quality metadata and gating

To account for variation in data completeness across paradigms, domain-level quality metadata were computed without access to outcome labels. For each domain, these included the within-domain missingness proportion and a binary availability indicator. These features parameterized a gating network that produced masked, normalized weights over domains, with unavailable domains assigned zero weight.

E3. Task heads

Two classification routes were evaluated.

a. Route A used a single three-class softmax head for NC, SD, and MDD classification.

b. Route B used a hierarchical two-step formulation. The first-stage head separated NC from the depression spectrum. The second-stage head separated SD from MDD among spectrum samples. Three-class probabilities were derived from the outputs of the two stages. For both routes, the predicted class was assigned according to the highest three-class probability. No post hoc decision-threshold optimization was performed.

A regression head estimated HAMD-17 as a continuous outcome using a robust regression loss.

E4. Training strategy

Model training followed a two-phase schedule, with an initial classification-only warm-up and a gradual increase in the regression-loss weight during joint optimization. Internal evaluation used five stratified outer train-test splits. Within each outer training set, stratified five-fold cross-validation generated out-of-fold logits for temperature scaling; models fitted within these folds used the corresponding validation fold for early stopping. The final model for each outer repeat was fitted using 90% of the outer training set, with a randomly selected 10% internal validation subset used for early stopping. Network architecture and training hyperparameters were fixed across outer repeats.

Imputation, outlier truncation, scaling, domain-quality computation, feature retention, model fitting, and calibration were estimated within the relevant training data. The fitted procedures were applied to validation and outer-test data without re-estimation. Performance therefore represents internal out-of-sample evaluation within the present cohort.

E5. Calibration

When reported, probability calibration used temperature scaling fitted on out-of-fold logits from the outer training data. Separate temperatures were estimated for the two classification stages in Route B.

E6. Feature-retention and domain-gating stability analyses

For the multi-task model, stability was assessed at the level of preprocessing-derived feature retention and task-domain composition because this model did not use the supervised LASSO/RF feature-selection procedure applied in the single-task models. Starting from the 723 raw eye-tracking features, retained features were recorded after missingness filtering, winsorization, low-variability filtering, and correlation-based redundancy reduction within each outer training-validation set. We summarized the number of retained features per outer repeat, persistent and robust retained features, pairwise Jaccard indices, retained task-domain blocks, and the largest task-domain blocks by retained feature count. These results are reported in Supplementary Tables S3, S14, and S15.

Domain-level gating weights were averaged within each outer repeat and then summarized across outer repeats for Route A and Route B. The five task-domain blocks with the highest mean gating weights within each route are shown in Supplementary Table S16. These weights were interpreted as relative domain-level contributions across task blocks, not as evidence of exclusive reliance on a single domain.

E7. Architecture-matched regression-only sensitivity analysis

To examine whether the classification objective influenced HAMD-17 estimation, we fitted an architecture-matched regression-only model. The outer splits, fold-wise preprocessing, task-domain definitions, domain encoders, domain-quality metadata, gating mechanism, fused representation, regression trunk, regression head, optimizer, and training limits were identical to those used for Route B. The classification-only warm-up was omitted and the classification losses were removed throughout training. Early stopping was based on validation regression performance. Outer-test MAE, RMSE, and R^2^ were compared with Route B within matched outer repeats (Supplementary Table S17).

**Supplementary Appendix F. Expanded statistical analysis and reporting details**

F1. Feature-level group comparisons

Baseline characteristics and eye-tracking features were analyzed using procedures appropriate for the distribution and measurement scale of each variable. Continuous baseline variables were compared across NC, SD, and MDD using one-way analysis of variance when model assumptions were satisfied and the Kruskal–Wallis test otherwise. Categorical variables were compared using the chi-square test or Fisher exact test, as appropriate. For eye-tracking features, group-level comparisons used the same distribution-guided framework. Effect sizes were reported as η² for analysis of variance and ε² for Kruskal–Wallis analyses. Features showing nominal group differences before multiple-testing correction were retained as candidate features for descriptive visualization, whereas formal inference was based on Benjamini–Hochberg false discovery rate correction across the tested eye-tracking feature set.

Covariate-adjusted analyses were performed for the 35 candidate features using age, sex, and BMI as prespecified covariates. These descriptive analyses reported nominal P values and were interpreted alongside the BH-FDR-corrected group comparisons, effect sizes, and group patterns.

The 539-feature testing family was defined without reference to diagnostic group, HAMD-17, group-comparison statistics, association P values, or effect sizes. BH-FDR correction was applied across the complete testing set.

F2. Linear, monotonic, and GAM-based associations with HAMD-17 severity

Associations between eye-tracking features and HAMD-17 total score were assessed using Pearson correlation for linear associations, Spearman correlation for monotonic associations, and penalized generalized additive models (GAMs) for smooth associations. GAMs were fitted separately for each feature in the complete retained eye-tracking feature set using a Gaussian identity-link model. The eye-tracking feature was entered as a penalized smooth term with a low basis dimension (k = 4). Each model used complete observations for the corresponding eye-tracking feature, HAMD-17, and covariates. No imputation was performed for the feature-level association analyses. For each analysis, Benjamini–Hochberg false discovery rate correction was applied across the complete testing family.

Robustness of the covariate-adjusted GAM associations was examined using stronger smoothing penalties (λ = 2 and λ = 5), with multiplicity correction applied separately across the complete feature set under each specification. Smooth-term effective degrees of freedom were summarized to characterize model flexibility. Incremental explanatory contribution was expressed as partial R^2^, defined as the reduction in residual sum of squares after addition of the eye-feature smooth relative to a covariate-only model.

Permutation-based sensitivity analysis used the Freedman–Lane procedure for associations meeting the primary FDR criterion. For each feature, a covariate-only model including age, sex, and body mass index was fitted, and its residuals were randomly permuted 5000 times while retaining the fitted covariate component. The full covariate-adjusted GAM was refitted to each permuted outcome, with partial R^2^ used as the permutation statistic. Empirical P values were calculated as (1 + number of permuted statistics at least as large as the observed statistic)/(5000 + 1), followed by BH-FDR correction across the permutation tests.

For descriptive curve characterization, fitted smooths were evaluated over the central 5th–95th percentile of the observed feature distribution. Overall direction and curve pattern were summarized from the fitted smooth and its numerical first derivative. These descriptors were not subjected to separate hypothesis testing and were not used to determine statistical significance.

F3. Predictive performance metrics

Classification performance was evaluated using accuracy, balanced accuracy, macro-averaged F1 score, weighted F1 score, macro-averaged precision, macro-averaged recall, macro-averaged one-versus-rest area under the receiver operating characteristic curve, and log loss. Accuracy and F1-based metrics were used to summarize threshold-dependent performance, whereas macro-AUROC and log loss were used to assess ranking ability and probabilistic fit. Confusion matrices were used to characterize diagnostic error patterns across NC, SD, and MDD.

Regression performance for HAMD-17 estimation was assessed using mean absolute error, mean squared error, root mean squared error, and R^2^. Mean absolute error was used as the primary clinically interpretable error metric, while R^2^ summarized explained variance in internal held-out test predictions.

Performance was summarized across five outer repeats. Summary tables report mean ± standard deviation, whereas Figures 1 and 2 show model-specific means with repeat-level 95% confidence intervals estimated using a t distribution with 4 degrees of freedom. Matched route, regression-only, and predictor-set comparisons used paired within-repeat differences. Because participants could appear in held-out sets across more than one repeat, these intervals characterize variability across resampling repeats rather than independent-sample uncertainty.

F4. Incremental predictive-value analyses

Incremental predictive value was assessed by comparing prespecified clinical-only models with combined models incorporating fold-wise selected eye-tracking features. Clinical predictors were organized into basic, lifestyle, and extended layers. Symptom scales used for eligibility screening, severity stratification, or outcome definition were excluded from predictor sets to reduce symptom-scale leakage. Tracking accuracy and tracking precision were also excluded from clinical predictor sets because they reflected eye-tracking data quality rather than clinical characteristics. Candidate models were ranked descriptively. No multiplicity-adjusted hypothesis testing was used for model ranking.

For classification, paired gains were calculated within matched outer test splits. For accuracy, balanced accuracy, F1, precision, recall, and macro-AUROC, gains were defined as combined-model performance minus clinical-only performance. For log loss, gains were defined as clinical-only log loss minus combined-model log loss, so positive values indicated improved probabilistic performance. For regression, gains in mean absolute error, mean squared error, and root mean squared error were defined as clinical-only error minus combined-model error, whereas R^2^ gains were defined as combined-model R^2^ minus clinical-only R^2^. Eye-tracking-only models were retained as reference models rather than paired comparators.

F5. Interpretability and stability analyses

Model interpretability analyses were used to characterize behavioral patterns contributing to prediction, without causal or mechanistic inference. Global SHAP analyses summarized feature contributions for classification and regression models, and LIME analyses provided local explanations for representative individual predictions. These analyses were interpreted descriptively and were considered exploratory.

Stability analyses were used to assess whether selected or retained eye-tracking features were consistent across outer repeats. For single-task classification and regression models, supervised feature-selection stability was summarized using the number of selected features per outer repeat, the number of unique features selected at least once, the number of features selected in all five repeats, and pairwise Jaccard indices. For the multi-task model, stability was assessed at the level of preprocessing-derived feature retention and task-domain composition because this model did not use the supervised LASSO/RF feature-selection procedure applied in the single-task models. Domain-level gating weights were summarized across outer repeats and interpreted as relative task-domain contributions rather than evidence of exclusive reliance on any single domain.

**Supplementary Appendix G. Reporting guideline checklists**

G1. STROBE checklist for cross-sectional studies

| **Section/topic** | **Item** | **Checklist requirement (concise)** | **Reported on page/section and status** |
| --- | --- | --- | --- |
| Title and abstract | 1a | State the observational design in the title or abstract. | **Reported.** Title (p. 1, lines 1–2); Abstract Methods (p. 3, lines 28–32). |
| Title and abstract | 1b | Provide a balanced summary of methods and findings. | **Reported.** Abstract (p. 3, lines 25–44). |
| Introduction | 2 | Explain the scientific background and rationale. | **Reported.** Introduction (p. 4, lines 47–65). |
| Introduction | 3 | State the objectives and any prespecified hypotheses. | **Reported.** Abstract Aims (p. 3, lines 26–27); Introduction (pp. 4–5, lines 66–72). |
| Methods | 4 | Present the main design features early. | **Reported.** Abstract Methods (p. 3, lines 28–32); Methods (pp. 5–8, lines 73–146). |
| Methods | 5 | Describe setting, locations, and relevant dates. | **Reported.** Methods 2.1 (p. 5, lines 75–88); Supplementary Appendix A6 (p. 2, lines 43–47). |
| Methods | 6a | Give eligibility criteria and participant sources/selection. | **Reported.** Methods 2.1 (p. 5, lines 75–89); Supplementary Appendices A1–A3 (pp. 1, lines 3–26). |
| Methods | 7 | Define outcomes, exposures/predictors, confounders, and diagnostic criteria. | **Reported.** Methods 2.1–2.6 (pp. 5–8, lines 74–147); Supplementary Appendices A and C–F (pp. 1–15, lines 1–428). |
| Methods | 8 | Describe data sources and measurement methods for each key variable. | **Reported.** Methods 2.1–2.3 (pp. 5–6, lines 74–102); Supplementary Appendices A–C (pp. 1–5, lines 1–133). |
| Methods | 9 | Describe measures used to address potential bias. | **Reported.** Methods 2.3–2.6 (pp. 6–8, lines 96–146); Supplementary Appendices C–F (pp. 4–15, lines 103–450). |
| Methods | 10 | Explain how the study size was determined. | **Reported.** Methods 2.1 (p. 5, lines 86–88); Limitations (p. 16, lines 324–329); Supplementary Appendix A6 (p. 2, lines 42–47). |
| Methods | 11 | Explain handling and categorisation of quantitative variables. | **Reported.** Methods 2.1–2.6 (pp. 5–8, lines 74–147); Supplementary Appendix A3 (p. 1, lines 15–26) and Appendix F (pp. 12–15, lines 335–428). |
| Methods | 12a | Describe statistical methods, including confounding control. | **Reported.** Methods 2.4–2.6 (pp. 6–8, lines 104–147); Supplementary Appendices D–F (pp. 5–15, lines 129–428). |
| Methods | 12b | Describe subgroup and interaction analyses. | **Not applicable.** No prespecified subgroup or interaction analyses were performed. |
| Methods | 12c | Explain handling of missing data. | **Reported.** Methods 2.3 (p. 6, lines 97–103) and 2.5 (pp. 7–8, lines 117–136); Supplementary Appendices C–E (pp. 4–12, lines 98–333). |
| Methods | 12d | Describe methods accounting for the sampling strategy, when applicable. | **Not applicable.** The study was not based on a probability-sampling survey. |
| Methods | 12e | Describe sensitivity analyses. | **Reported.** Methods 2.4 (pp. 6–7, lines 104–116); Results 3.4 (pp. 9–10, lines 176–189) and 3.7 (pp. 12–13, lines 240–260); Supplementary Tables S2B, S2C, and S17. |
| Results | 13a | Report participant numbers at each stage. | **Partly reported.** Results 3.1 (pp. 8–9, lines 149–161) and Table 1 (pp. 24–29) report the analytic cohort; screening and eligibility-stage counts are not reported. |
| Results | 13b | Give reasons for non-participation at each stage. | **Not reported.** Reasons for non-participation or exclusion before entry into the analytic cohort are not provided. |
| Results | 13c | Consider a participant-flow diagram. | **Not reported.** Supplementary Figure S1 presents analysis pathways and is not a participant-flow diagram. |
| Results | 14a | Report participant characteristics and potential confounders. | **Reported.** Results 3.1 (pp. 8–9, lines 149–161); Table 1 (pp. 24–29). |
| Results | 14b | Report missing data for each variable of interest. | **Partly reported.** Methods 2.3 (p. 6, lines 97–103) and Supplementary Appendix C (pp. 4–5, lines 98–127) describe eye-tracking missingness; baseline variable-level missingness is not tabulated. |
| Results | 15 | Report outcome data or summary measures. | **Reported.** Results 3.3–3.7 (pp. 9–13, lines 166–260); Table 1 (pp. 24–29); Figure 1–5 legends (pp. 30–31, lines 459–499); Supplementary Tables S1–S17. |
| Results | 16a | Report unadjusted and adjusted estimates with precision, and identify confounders. | **Reported.** Results 3.3–3.7 (pp. 9–13, lines 166–260); covariates are specified in Methods 2.4 (p. 6, lines 110–113); Supplementary Tables S1–S2, S9, S13, and S17. |
| Results | 16b | Report category boundaries for categorised continuous variables. | **Reported.** Methods 2.1 (p. 5, lines 75–85); Supplementary Appendix A3 (pp. 1–2, lines 15–26). |
| Results | 16c | Translate relative risks into absolute risks when relevant. | **Not applicable.** The study did not estimate longitudinal relative risks. |
| Results | 17 | Report other analyses, including sensitivity analyses. | **Reported.** Results 3.4–3.7 (pp. 9–13, lines 176–260); Supplementary Tables S2B, S2C, S9, S13, and S17. |
| Discussion | 18 | Summarise key results in relation to objectives. | **Reported.** Discussion (pp. 13–17, lines 261–349). |
| Discussion | 19 | Discuss limitations, potential bias, and imprecision. | **Reported.** Limitations (pp. 16–17, lines 324–349). |
| Discussion | 20 | Provide a cautious interpretation considering objectives, limitations, and other evidence. | **Reported.** Discussion (pp. 13–17, lines 261–349). |
| Discussion | 21 | Discuss generalisability/external validity. | **Reported.** Discussion and Limitations (pp. 15–17, lines 300–349). |
| Other information | 22 | Report funding sources and funder roles. | **Reported.** Funding statement (p. 1, lines 16–20). |

G2. TRIPOD+AI checklist for prediction-model studies

| **Section/topic** | **Item** | **Applies** | **Checklist requirement (concise)** | **Reported on page/section and status** |
| --- | --- | --- | --- | --- |
| Title | 1 | D/E | Identify model development/evaluation, target population, and predicted outcome. | **Partly reported.** The title identifies the population and outcomes (p. 1, lines 1–2); development and internal evaluation are stated in the Abstract (p. 3, lines 26–45) and Methods 2.5–2.6 (pp. 7–8, lines 117–147). |
| Abstract | 2 | D/E | Report the abstract using the TRIPOD+AI for Abstracts items. | **Partly reported.** See the completed TRIPOD+AI for Abstracts table below. |
| Introduction | 3a | D/E | Explain the healthcare context and rationale, including existing models. | **Reported.** Introduction (pp. 4–5, lines 48–66). |
| Introduction | 3b | D/E | Describe the target population, intended purpose, care pathway, and users. | **Reported.** Introduction (pp. 4–5, lines 54–73) and Discussion (p. 15, lines 300–311). |
| Introduction | 3c | D/E | Describe known sociodemographic health inequalities relevant to the target population. | **Not reported.** Relevant health inequalities and demographic performance disparities are not discussed. |
| Introduction | 4 | D/E | State objectives and whether the study develops, evaluates, or both. | **Reported.** Introduction (p. 5, lines 67–73); Methods 2.5–2.6 (pp. 7–8, lines 117–147). |
| Methods: Data | 5a | D/E | Describe data sources, rationale, and representativeness for development/evaluation. | **Reported.** Methods 2.1 (p. 5, lines 75–89); Supplementary Appendix A (pp. 1–2, lines 2–51); representativeness limitations (pp. 16–17, lines 324–349). |
| Methods: Data | 5b | D/E | Give dates of participant accrual and follow-up when applicable. | **Reported.** Supplementary Appendix A6 (p. 2, lines 42–47). Follow-up is not applicable to this cross-sectional study. |
| Methods: Participants | 6a | D/E | Describe setting, number of centres, and locations. | **Reported.** Methods 2.1 (p. 5, lines 76–77). |
| Methods: Participants | 6b | D/E | Describe eligibility criteria. | **Reported.** Methods 2.1 (p. 5, lines 76–85); Supplementary Appendices A1–A3 (pp. 1–2, lines 4–29). |
| Methods: Participants | 6c | D/E | Describe relevant treatments and how they were handled. | **Reported.** Supplementary Appendix A5 (p. 2, lines 35–41); Limitations (pp. 16–17, lines 332–338). |
| Methods: Data preparation | 7 | D/E | Describe preprocessing and quality checks, including demographic consistency when relevant. | **Reported.** Methods 2.3 and 2.5 (pp. 6–8, lines 98–103 and 117–136); Supplementary Appendices C–E (pp. 4–12, lines 98–333). Demographic-specific preprocessing comparisons were not performed. |
| Methods: Outcome | 8a | D/E | Define each predicted outcome, timing, rationale, and assessment method. | **Reported.** Methods 2.1 and 2.6 (pp. 5 and 7–8, lines 76–85 and 137–147); same-day assessment is stated at p. 5, lines 84–85. |
| Methods: Outcome | 8b | D/E | Describe qualifications and demographic characteristics of subjective outcome assessors. | **Partly reported.** Assessor qualifications and procedures are described in Methods 2.1 (p. 5, lines 77–86) and Supplementary Appendix A4 (pp. 1–2, lines 27–34); assessor demographics are not reported. |
| Methods: Outcome | 8c | D/E | Describe blinding of outcome assessment to predictors. | **Reported.** Supplementary Appendix A4 (pp. 1–2, lines 27–34). |
| Methods: Predictors | 9a | D | Explain selection of initial predictors and any preselection. | **Reported.** Methods 2.2–2.5 (pp. 5–8, lines 90–136); Supplementary Appendices B–D (pp. 2–10, lines 53–264). |
| Methods: Predictors | 9b | D/E | Define predictors and describe how and when they were measured. | **Reported.** Methods 2.2–2.3 (pp. 5–6, lines 90–103); Supplementary Appendices B–C (pp. 2–5, lines 53–127). |
| Methods: Predictors | 9c | D/E | Describe qualifications of assessors when predictor measurement is subjective. | **Not applicable.** Eye-tracking predictors were generated from instrument-recorded samples using prespecified algorithms. |
| Methods: Sample size | 10 | D/E | Explain and justify the study size, including calculations when available. | **Reported.** Methods 2.1 (p. 5, lines 75–89); Limitations (p. 16, lines 324–330); Supplementary Appendix A6 (p. 2, lines 42–47). No formal a priori calculation was performed. |
| Methods: Missing data | 11 | D/E | Describe missing-data handling and reasons for data omission. | **Reported.** Methods 2.3 and 2.5 (pp. 6–8, lines 97–103 and 118–136); Supplementary Appendices C–E (pp. 4–12, lines 98–333). |
| Methods: Analysis | 12a | D | Explain how data were partitioned for development and performance evaluation. | **Reported.** Methods 2.5–2.6 (pp. 7–8, lines 117–143); Supplementary Appendices D1 and E4 (pp. 5 and 11, lines 135–147 and 310–322). |
| Methods: Analysis | 12b | D | Describe transformations, scaling, and predictor handling. | **Reported.** Methods 2.5 (p. 7, lines 117–123); Supplementary Appendix C and D2 (pp. 4–6, lines 98–151). |
| Methods: Analysis | 12c | D | Specify model types, rationale, building steps, tuning, and internal validation. | **Reported.** Methods 2.5–2.6 (pp. 7–8, lines 124–147); Supplementary Appendices D–E (pp. 5–12, lines 129–333); Supplementary Tables S6–S7 and S11. |
| Methods: Analysis | 12d | D/E | Describe handling and quantification of cluster/site heterogeneity. | **Reported.** Site balance and acquisition quality were assessed in Methods 2.4 (p. 6, lines 104–108) and Results 3.1–3.2 (pp. 8–9, lines 149–165). Site-specific prediction performance was not estimated (Limitations, p. 16, lines 324–330). |
| Methods: Analysis | 12e | D/E | Specify performance measures and plots and how models were compared. | **Reported.** Methods 2.5–2.6 (pp. 7–8, lines 117–147); Supplementary Appendices F3–F4 (pp. 14–15, lines 380–413); Figure 1–5 legends (pp. 30–31, lines 459–499). |
| Methods: Analysis | 12f | E | Describe model updating following evaluation. | **Not applicable.** No external evaluation or model updating was performed. |
| Methods: Analysis | 12g | E | Explain how predictions were generated during evaluation. | **Reported.** Supplementary Appendices D4–D5 and E3–E5 (pp. 6–11, lines 161–195 and 284–309); analytical code is available on request (p. 1, lines 13–15). |
| Methods: Class imbalance | 13 | D/E | Describe class-imbalance methods when used. | **Not applicable.** The groups were balanced at 61 participants each (Results 3.1, p. 8, lines 149–153); no class-imbalance correction was used. |
| Methods: Fairness | 14 | D/E | Describe methods used to assess or address model fairness. | **Reported as not performed.** Subgroup fairness analyses were not feasible at this sample size (Limitations, p. 16, lines 324–330). |
| Methods: Model output | 15 | D | Describe model outputs, classification rules, and threshold selection. | **Reported.** Supplementary Appendices D4 and E3 (pp. 6 and 11, lines 162–178 and 284–292). Labels were assigned by highest class probability without post hoc threshold optimization. |
| Methods: Training vs evaluation | 16 | D/E | Identify differences between development and evaluation data. | **Reported.** Methods 2.5 (pp. 7–8, lines 118–136). Development and held-out evaluation sets were stratified splits from the same cohort. |
| Methods: Ethics | 17 | D/E | Name the ethics committee and describe consent or waiver. | **Reported.** Ethics approval statement (pp. 1–2, lines 22–25). |
| Open science | 18a | D/E | Report funding sources and funder roles. | **Reported.** Funding statement (p. 1, lines 16–20). |
| Open science | 18b | D/E | Declare conflicts and financial disclosures. | **Reported.** Conflict of interest disclosure (p. 1, line 21). |
| Open science | 18c | D/E | State where the protocol can be accessed or that none was prepared. | **Reported.** No formal prospective prediction-model protocol was publicly archived (Supplementary Appendix A7, p. 2, lines 48–51). |
| Open science | 18d | D/E | Give registration details or state that the study was not registered. | **Reported.** The study was not registered (Supplementary Appendix A7, p. 2, lines 48–51). |
| Open science | 18e | D/E | Describe data availability. | **Reported.** Data availability statement (p. 1, lines 13–15). |
| Open science | 18f | D/E | Describe analytical-code availability. | **Reported.** Data availability statement (p. 1, lines 13–15). |
| Patient/public involvement | 19 | D/E | Describe patient/public involvement or state that there was none. | **Reported.** No patient or public involvement (Supplementary Appendix A7, p. 2, lines 48–51). |
| Results: Participants | 20a | D/E | Describe participant flow and outcome distribution. | **Partly reported.** Results 3.1 (pp. 8–9, lines 149–161) and Table 1 (pp. 24–29) report the analytic cohort; screening and exclusion-stage counts are not reported. |
| Results: Participants | 20b | D/E | Report participant characteristics, predictors, treatments, sample size, outcomes, and missingness. | **Reported.** Results 3.1–3.2 (pp. 8–9, lines 149–165); Table 1 (pp. 24–29); Supplementary Appendix A5 (p. 2, lines 35–41) and Appendices C–E (pp. 4–12, lines 98–333). |
| Results: Participants | 20c | E | Compare evaluation data with development data. | Not applicable to an external dataset. Evaluation subsets were stratified held-out splits from the same cohort (Methods 2.5, pp. 7–8, lines 118–136). |
| Results: Model development | 21 | D/E | Report participant and outcome numbers in each modelling analysis. | **Partly reported.** Cohort size and group counts are given in Methods 2.1 and Results 3.1 (pp. 5 and 8, lines 87–89 and 149–153), and the 85%/15% split in Methods 2.5 (p. 7, lines 118–119); fold-specific counts are not tabulated. |
| Results: Model specification | 22 | D | Provide the full model or implementation resource for new predictions. | **Partly reported.** Architecture and training details are in Supplementary Appendix E (pp. 10–12, lines 266–333), and code is available on request (p. 1, lines 13–15); no deployable model object is provided. |
| Results: Model performance | 23a | D/E | Report performance with confidence intervals, including key subgroups where possible. | **Reported for overall performance.** Results 3.5–3.7 (pp. 10–13, lines 190–260), Figures 1–4 (pp. 30–31, lines 459–492), and Supplementary Tables S9, S13, and S17 report repeat-level 95% CIs; subgroup performance is not reported. |
| Results: Model performance | 23b | D/E | Report heterogeneity in performance across clusters when examined. | **Not applicable.** Site-specific performance heterogeneity was not examined (Limitations, p. 16, lines 324–330). |
| Results: Model updating | 24 | E | Report results of model updating. | **Not applicable.** No model updating was performed. |
| Discussion | 25 | D/E | Interpret results, including fairness considerations, in relation to objectives and prior studies. | **Partly reported.** Interpretation is provided in Discussion (pp. 13–17, lines 262–349); fairness was not evaluated (p. 16, lines 329–330). |
| Discussion | 26 | D/E | Discuss limitations and effects on bias, uncertainty, and generalisability. | **Reported.** Limitations (pp. 16–17, lines 324–349). |
| Discussion: Usability | 27a | D | Explain handling of poor-quality or unavailable input data in implementation. | **Reported for the research pipeline.** Supplementary Appendix E2 (p. 10, lines 278–283) describes availability masks and gating; Appendices C–D (pp. 4–10, lines 98–264) describe missing-data handling. |
| Discussion: Usability | 27b | D | Describe required user interaction and expertise. | Not applicable to a deployed system. No clinical implementation or user interface was developed. |
| Discussion: Future work | 27c | D/E | State next research steps for applicability and generalisability. | **Reported.** Discussion and Summary (pp. 15–17, lines 308–311 and 344–354). |

TRIPOD+AI for Abstracts

| **Section** | **Item** | **Checklist requirement (concise)** | **Reported in current abstract and status** |
| --- | --- | --- | --- |
| Title | 1 | Identify model development/evaluation, target population, and predicted outcome. | **Partly reported.** Title (p. 1, lines 1–2) identifies the depressive spectrum and joint diagnostic/severity modeling; development and evaluation wording is implicit. |
| Background | 2 | Briefly explain the healthcare context and rationale. | **Partly reported.** Abstract Aims (p. 3, lines 27–28); full context in Introduction (pp. 4–5, lines 48–73). |
| Objectives | 3 | State whether the study develops, evaluates, or both. | **Reported.** Abstract Aims and Methods (p. 3, lines 27–33). |
| Methods | 4 | Describe the data source. | **Partly reported.** Abstract Methods (p. 3, lines 29–33) states the cross-sectional design; recruitment sites are in Methods 2.1 (p. 5, lines 76–77). |
| Methods | 5 | Describe eligibility criteria and setting. | Not reported in the abstract because of the 200-word limit; reported in Methods 2.1 (p. 5, lines 76–89) and Supplementary Appendix A (pp. 1–2, lines 2–51). |
| Methods | 6 | Specify the predicted outcomes and time horizon when relevant. | **Reported.** Abstract Methods and Results (p. 3, lines 29–42); a prognostic time horizon is not applicable. |
| Methods | 7 | Summarise model type, building steps, and internal validation. | **Partly reported.** Abstract Methods (p. 3, lines 29–33) states the model classes, repeated nested resampling, and fold-specific preprocessing. |
| Methods | 8 | State performance measures. | **Partly reported.** Accuracy, macro F1, and R2 appear in Abstract Results (p. 3, lines 38–41); all measures are specified in Methods 2.5–2.6 (pp. 7–8, lines 117–147). |
| Results | 9 | Report participant and outcome numbers. | **Partly reported.** Total n=183 is given in Abstract Methods (p. 3, line 29); group counts are in Results 3.1 (p. 8, lines 149–153). |
| Results | 10 | Summarise final predictors. | **Reported at a high level.** Abstract Results names the FDR-significant feature domains (p. 3, lines 34–38). |
| Results | 11 | Report performance estimates with confidence intervals. | **Partly reported.** Point estimates are given in Abstract Results (p. 3, lines 38–41); confidence intervals are omitted because of the 200-word limit. |
| Discussion | 12 | Provide an overall interpretation. | **Reported.** Abstract Conclusion (p. 3, lines 42–45). |
| Registration | 13 | Give registry details. | **Reported outside the abstract.** The study was not registered (Supplementary Appendix A7, p. 2, lines 48–51); registration was omitted from the abstract because of the journal word limit. |

**Supplementary Figure S1. Eye-tracking preprocessing and analysis pathways**

The participant-level eye-tracking matrix comprised 723 variables and entered two analytically distinct pathways. Feature-level inferential analyses used a label-blind quality-control and redundancy-reduction procedure based on missingness, variability, and inter-feature correlation, yielding 539 variables for group comparisons and HAMD-17 association analyses. Diagnostic group, HAMD-17 score, P values, and effect sizes did not contribute to this reduction. BH-FDR correction was applied across the relevant testing family. Predictive modeling began from the 723-variable matrix within each outer training–validation set. Label-blind preprocessing was estimated from the corresponding training data. Single-task models subsequently used outcome-guided feature selection within inner cross-validation, whereas the multi-task model used preprocessing-derived feature retention without a supervised feature-selection step. Inner folds supported temperature calibration for the multi-task model, and an internal validation subset guided early stopping. Fitted procedures were applied unchanged to held-out outer-test data. Held-out outer-test outcomes did not inform preprocessing, feature selection, model tuning, calibration, early stopping, or ensemble fitting. Abbreviations: BH-FDR, Benjamini–Hochberg false discovery rate; HAMD-17, 17-item Hamilton Depression Rating Scale.

**Supplementary Figure S2. Spatial distribution of gaze during lateral gaze and smooth pursuit tasks**

Relative fixation-density maps are shown for NC, SD, and MDD during the lateral gaze task and smooth pursuit tasks with color interference. Panels A to C show the lateral gaze task for NC, SD, and MDD. Panels D to F show constant-velocity smooth pursuit with color interference for NC, SD, and MDD. Panels G to I show sinusoidal smooth pursuit with color interference for NC, SD, and MDD. Warmer colors indicate higher fixation density. Abbreviations: NC, normal control; SD, subthreshold depression; MDD, major depressive disorder.

**Supplementary Figure S3. Spatial distribution of attention to facial stimuli across emotional valence conditions**

Relative fixation-density maps are shown for attention to facial stimuli under positive, neutral, and negative valence conditions. Panels A to C show positive faces for NC, SD, and MDD. Panels D to F show neutral faces for NC, SD, and MDD. Panels G to I show negative faces for NC, SD, and MDD. Warmer colors indicate higher fixation density within facial AOIs. Abbreviations: NC, normal control; SD, subthreshold depression; MDD, major depressive disorder; AOI, area of interest.

**Supplementary Figure S4. Top 20 covariate-adjusted eye-tracking features across diagnostic groups**

Violin plots show feature distributions across the three diagnostic groups. Features are ordered by ηp^2^ from covariate-adjusted group models including age, sex, and body mass index. In each violin plot, the central line indicates the median and the inner band indicates the interquartile range. Panel titles show the feature name and ηp². The adjusted P value for the overall group effect is shown in each panel. Abbreviations: NC, normal control; SD, subthreshold depression; MDD, major depressive disorder; AOI, area of interest; ηp², adjusted partial eta-squared.

**Supplementary Figure S5. Covariate-adjusted GAM associations for the eight FDR-significant eye-tracking features identified across all 539 retained features**

Fitted smooths are shown for the eight eye-tracking features that met the BH-FDR criterion in the primary covariate-adjusted GAM analysis. Solid lines represent the estimated smooth terms from models adjusted for age, sex, and BMI, and shaded bands indicate pointwise 95% confidence intervals. Rug marks along the x-axis indicate the distribution of observed feature values. Smooths are displayed over the 1st–99th percentile of each feature's observed distribution. Panel annotations report the BH-FDR-adjusted q value from the complete 539-feature testing family and adjusted partial R^2^, defined as the incremental reduction in residual sum of squares relative to the covariate-only model. The plotted curves cover the 1st to 99th percentiles. Descriptive curve classifications in Supplementary Table S2B were based on the central 5th to 95th percentiles. Abbreviations: GAM, generalized additive model; HAMD-17, 17-item Hamilton Depression Rating Scale; AOI, area of interest; BMI, body mass index; BH-FDR, Benjamini–Hochberg false discovery rate.

**Supplementary Figure S6. Supervised eye-tracking feature selection for three-class classification in outer repeat 1**

Panels A and B show the features retained by L1-regularized multinomial logistic regression and ranked by random forest permutation importance, respectively, within the outer training–validation set of outer repeat 1. Panel C summarizes the overlap between the two selectors and their union, and Panel D lists features retained by both methods. The displayed feature counts apply to this outer repeat and were used only for model development within the corresponding outer training–validation partition. Feature-selection stability across all five outer repeats is reported in Supplementary Tables S3–S5. Abbreviations: AOI, area of interest; RF, random forest.

**Supplementary Figure S7. Base-learner weight distributions across outer repeats for the NNLS Super Learner and softmax-weighted ensembles**

Panels A and B show boxplots of base-learner weights across five outer repeats for the NNLS Super Learner and the softmax-weighted ensemble, respectively. Higher weights indicate greater contribution to ensemble prediction. Panels C and D show summary weights across repeats, presented as mean ± standard deviation. The softmax panels include only base learners that entered the Top-k set in at least one outer repeat. The NNLS panels include all candidate base learners, with most weights close to zero. Abbreviations: NNLS, non-negative least squares.

**Supplementary Figure S8. Outer-test performance of ensemble models relative to the mean-averaging baseline**

For ACC, BACC, F1, precision, recall, and macro-AUROC, values are presented as percentage-point differences relative to the mean-averaging ensemble, with positive values indicating improved performance. For log loss, values are presented as percentage reduction relative to mean averaging, with positive values indicating improvement. The table summarizes outer-test performance across five repeats as mean ± standard deviation. SL denotes SuperLearner-NNLS, Stack denotes Stacking-LogReg, and Soft denotes softmax weighting. Abbreviations: ACC, accuracy; BACC, balanced accuracy; AUROC, area under the receiver operating characteristic curve; NNLS, non-negative least squares.

**Supplementary Figure S9. Confusion matrices of SuperLearner-NNLS across five outer repeats and the mean matrix in the eye-tracking modality**

Each panel shows the confusion matrix from one outer-repeat test set. The bottom-right panel shows the mean confusion matrix across repeats, rounded to the nearest integer. Rows denote true labels and columns denote predicted labels. Cells report the number of participants and the within-row percentage. Abbreviations: NC, normal control; SD, subthreshold depression; MDD, major depressive disorder; NNLS, non-negative least squares.

**Supplementary Figure S10. Global SHAP feature contributions for the SuperLearner-NNLS ensemble in the eye-tracking modality**

The top 20 features are ranked according to the mean absolute SHAP value aggregated across the three classes. Panel A shows class-specific SHAP importance for NC, SD, and MDD. Panel B shows stacked contributions across classes to summarize overall feature importance. These summaries reflect global contribution patterns rather than individual-level explanations and include AOI-derived features where applicable. Abbreviations: SHAP, Shapley additive explanations; NC, normal control; SD, subthreshold depression; MDD, major depressive disorder; AOI, area of interest; NNLS, non-negative least squares.

**Supplementary Figure S11. SHAP beeswarm and dependence plots for the SD class in the eye-tracking modality**

The left panel shows SHAP value distributions for the 20 features most relevant to the SD class, with percentages indicating each feature’s share of the total mean absolute SHAP value within the top-20 set. The right panels show dependence plots for selected features, relating feature values to SHAP contributions for the SD output and illustrating overlap with the NC and MDD classes. AOI-derived features are included where applicable. Abbreviations: SHAP, Shapley additive explanations; NC, normal control; SD, subthreshold depression; MDD, major depressive disorder; AOI, area of interest.

**Supplementary Figure S12. Individual-level LIME explanations for SuperLearner-NNLS predictions in the eye-tracking modality**

Two representative participants are shown for each class. Each panel title reports the predicted class and the corresponding predicted probability. Bars indicate the direction and magnitude of local contributions from discretized feature intervals, including AOI-derived features where applicable, with positive values increasing and negative values decreasing the predicted probability for the displayed class. Abbreviations: LIME, local interpretable model-agnostic explanations; NC, normal control; SD, subthreshold depression; MDD, major depressive disorder; AOI, area of interest.

**Supplementary Figure S13. Supervised eye-tracking feature selection for HAMD-17 regression in outer repeat 1**

Panels A and B show the features retained by LASSO and ranked by random forest permutation importance, respectively, within the outer training–validation set of outer repeat 1. Panel C summarizes the overlap between the two selectors and their union, and Panel D lists features retained by both methods. The displayed feature counts apply to this outer repeat and were used only for model development within the corresponding outer training–validation partition. Feature-selection stability across all five outer repeats is reported in Supplementary Tables S3 and S10. Abbreviations: AOI, area of interest; HAMD-17, 17-item Hamilton Depression Rating Scale; LASSO, least absolute shrinkage and selection operator; RF, random forest.

**Supplementary Figure S14. Base-learner weight profiles and across-repeat stability of ensemble models for HAMD-17 regression in the eye-tracking modality**

Heat maps show base-learner weights across five outer repeats for SuperLearner-NNLS, softmax weighting, and stacking-ridge. Bar plots summarize weights across repeats as mean ± standard deviation and are shown for models with the largest contributions. Abbreviations: NNLS, non-negative least squares; HAMD-17, 17-item Hamilton Depression Rating Scale.

**Supplementary Figure S15. Outer-test performance differences between learning-based ensembles and mean averaging for HAMD-17 regression**

The upper panel shows percent improvement relative to the mean-averaging ensemble. Reductions in MAE, MSE, and RMSE indicate better performance, whereas increases in R^2^ indicate improved model fit. The lower panel summarizes outer-test performance across five repeats as mean ± SD. NNLS-based and other learning-based ensembles are shown for comparison. Abbreviations: HAMD-17, 17-item Hamilton Depression Rating Scale; MAE, mean absolute error; MSE, mean squared error; RMSE, root mean squared error; R^2^, coefficient of determination; NNLS, non-negative least squares.

**Supplementary Figure S16. Agreement between observed and predicted HAMD-17 scores on outer test sets across ensemble strategies**

Scatter plots show observed versus predicted HAMD-17 values for outer-test samples. The dashed line indicates the line of identity. Solid lines indicate linear fitted trends, and shaded bands indicate uncertainty. Marginal histograms summarize the distributions of observed and predicted scores. Performance summaries include R^2^, MAE, and RMSE, with NNLS-based ensemble predictions shown where applicable. Abbreviations: HAMD-17, 17-item Hamilton Depression Rating Scale; NNLS, non-negative least squares; R^2^, coefficient of determination; MAE, mean absolute error; RMSE, root mean squared error.

**Supplementary Figure S17. Global SHAP feature importance and effect patterns for the eye-tracking regression model**

The left panel shows the top 20 features ranked by mean absolute SHAP value, with beeswarm distributions summarized across samples. Percentages indicate each feature’s share of the total mean absolute SHAP value within the top-20 set. The right panels show dependence plots for the six most influential features, relating feature values to SHAP contributions. Point color reflects HAMD-17 level, and AOI-derived features are included where applicable. Abbreviations: AOI, area of interest; SHAP, Shapley additive explanations; HAMD-17, 17-item Hamilton Depression Rating Scale.

**Supplementary Figure S18. Individual-level LIME explanations for HAMD-17 regression across severity tertiles**

Two representative participants are shown from each HAMD-17 tertile defined by cut points q1 = 7 and q2 = 16. Bars represent local feature contributions within the neighborhood of each participant, including AOI-derived features where applicable, with positive values increasing and negative values decreasing the predicted score. Each panel reports the observed and predicted HAMD-17 values together with the feature-interval terms showing the largest contributions. Abbreviations: AOI, area of interest; LIME, local interpretable model-agnostic explanations; HAMD-17, 17-item Hamilton Depression Rating Scale.

**Supplementary Figure S19. Residual diagnostics for HAMD-17 prediction under Route A and Route B**

Panels A and C correspond to Route A, and panels B and D correspond to Route B. Panels A and B show Bland–Altman plots of prediction error, defined as predicted minus observed, against the mean of predicted and observed values. Dashed lines indicate the mean bias and the 95% limits of agreement. Panels C and D show residuals versus fitted predicted HAMD-17 values, with the zero-residual reference line. Each point represents one out-of-sample prediction from the outer test splits. Abbreviations: HAMD-17, 17-item Hamilton Depression Rating Scale.
